# Supplementary material for: Antibody levels following vaccination against SARS-CoV-2: associations with post-vaccination infection and risk factors in two UK longitudinal studies
Source: eLife. 2023 Jan 24;12:e80428. doi: 10.7554/eLife.80428 (PMC9940912; doi:10.7554/eLife.80428)
Supplement: Supplementary file 2. — Individuals are stratified by vaccination status at time of sampling. Data shown for individuals sampled at least 4 (2) weeks after first (second or third) vaccination. The antibody level assay range is 0.4–250 BAU/mL for Q2 results and 0.4–25000 BAU/mL for Q4 results, with a positive threshold of 0.8 BAU/mL. Categories with fewer than five individuals are suppressed. [file elife-80428-supp2.docx]

Supplementary file 2. Anti-Spike antibody level values and characteristics for individuals from TwinsUK sampled in Q2 and Q4 antibody collections. Individuals are stratified by vaccination status at time of sampling. Data shown for individuals sampled at least 4 (2) weeks after first (second or third) vaccination. The antibody level assay range is 0.4 to 250 BAU/mL for Q2 results and 0.4 to 25,000 BAU/mL for Q4 results, with a positive threshold of 0.8 BAU/mL. Categories with fewer than 5 individuals are suppressed.

| **Cohort** | **TwinsUK** | | | | | | | **ALSPAC** | | | |
| --- | --- | --- | --- | --- | --- | --- | --- | --- | --- | --- | --- |
| **Testing period** | **Q2** | **Q4** | **Q2** | | | **Q4** | | **Q2** | | | |
| **Vaccination status** | **All results** | **All results** | **Not vaccinated** | **Single-vaccinated** | **Double-vaccinated** | **Double-vaccinated** | **Triple-vaccinated** | **All results** | **Not vaccinated** | **Single-vaccinated** | **Double-vaccinated** |
| n | 4256 | 3575 | 330 | 1375 | 748 | 691 | 1937 | 1779 | 36 | 1459 | 284 |
| Anti-Spike antibody level value (BAU/mL): Median (IQR) | 80.78 (18.55, 250.0) | 10403.0 (3510.0, 20224.0) | 0.4 (0.4, 0.4) | 53.3 (22.72, 121.2) | 250.0 (250.0, 250.0) | 1317.0 (337.0, 5202.5) | 13694.0 (8153.0, 23543.0) | 58.93 (21.25, 247.5) | 10.53 (0.4, 48.69) | 43.42 (17.98, 106.65) | 250.0 (250.0, 250.0) |
| Anti-Spike antibody level value (BAU/mL): 5th & 10th percentile | 0.4, 0.4 | 134.6, 401.4 | 0.4, 0.4 | 4.58, 9.59 | 152.83, 250.0 | 73.7, 112.0 | 3446.0, 5036.0 | 3.19, 7.28 | 0.4, 0.4 | 3.2, 6.51 | 155.61, 250.0 |
| Anti-Spike antibody status: Positive, n (%) | 3372/3912 (86.2%) | 3423/3445 (99.4%) | 79/330 (23.9%) | 1357/1375 (98.7%) | 745/748 (99.6%) | 690/691 (99.9%) | 1936/1937 (99.9%) | 1745/1779 (98.1%) | 23/36 (63.9%) | 1440/1459 (98.7%) | 282/284 (99.3%) |
| Age (years): Median (IQR) | 63.0 (49.0, 72.0) | 63.0 (51.0, 72.0) | 38.0 (31.0, 44.0) | 63.0 (56.0, 69.0) | 70.0 (56.0, 77.0) | 49.0 (38.0, 59.0) | 69.0 (60.0, 74.0) | 60.0 (57.0, 62.0) | 57.5 (52.75, 62.25) | 60.0 (57.0, 63.0) | 59.0 (56.0, 61.0) |
| Sex: Male, n (%) | 518/4255 (12.2%) | 447/3574 (12.5%) | 48/330 (14.5%) | 178/1375 (12.9%) | 88/748 (11.8%) | 103/691 (14.9%) | 225/1937 (11.6%) | 451/1779 (25.4%) | 8/36 (22.2%) | 397/1459 (27.2%) | 46/284 (16.2%) |
| Ethnicity: Other than White, n (%) | 118/4219 (2.8%) | 96/3536 (2.7%) | 16/329 (4.9%) | 29/1368 (2.1%) | 19/739 (2.6%) | 26/686 (3.8%) | 39/1914 (2.0%) | 26/1779 (1.5%) | < 5 | 20/1459 (1.4%) | 5/284 (1.8%) |
| Local area deprivation, IMD decile: Median (IQR) | 7.0 (5.0, 9.0) | 7.0 (5.0, 9.0) | 7.0 (5.0, 9.0) | 8.0 (5.0, 9.0) | 7.0 (5.0, 9.0) | 7.0 (5.0, 9.0) | 8.0 (5.0, 9.0) |  |  |  |  |
| Local area deprivation, IMD: Most deprived 40% (decile 1-4), n (%) | 766/4242 (18.1%) | 640/3564 (18.0%) | 81/330 (24.5%) | 214/1371 (15.6%) | 124/748 (16.6%) | 151/690 (21.9%) | 298/1933 (15.4%) | 384/1213 (31.7%) | 9/24 (37.5%) | 305/971 (31.4%) | 70/218 (32.1%) |
| Highest educational attainment: NVQ level 3 or lower, n (%) | 1472/3493 (42.1%) | 1253/3025 (41.4%) | 49/224 (21.9%) | 497/1206 (41.2%) | 275/652 (42.2%) | 171/525 (32.6%) | 763/1732 (44.1%) | 1176/1694 (69.4%) | 30/36 (83.3%) | 936/1388 (67.4%) | 210/270 (77.8%) |
| Zygosity: Monozygotic, n (%) | 2722/4253 (64.0%) | 2280/3573 (63.8%) | 248/328 (75.6%) | 883/1375 (64.2%) | 459/748 (61.4%) | 490/689 (71.1%) | 1170/1937 (60.4%) |  |  |  |  |
| Weeks since first vaccination: Median (IQR) | 10.0 (6.0, 12.0) | 42.0 (38.0, 45.0) | -5.0 (-8.0, -3.0) | 8.0 (6.0, 9.0) |  |  |  |  |  | 6.0 (5.0, 8.0) |  |
| First vaccination received: AZD1222, n (%) | 2124/3591 (59.1%) | 1980/3378 (58.6%) | 70/275 (25.5%) | 1103/1374 (80.3%) |  |  |  |  |  | 1235/1459 (84.6%) |  |
| First vaccination received: BNT162b2, n (%) | 1410/3591 (39.3%) | 1336/3378 (39.6%) | 170/275 (61.8%) | 266/1374 (19.4%) |  |  |  |  |  | 224/1459 (15.4%) |  |
| First vaccination received: Other, n (%) | 57/3591 (1.6%) | 62/3378 (1.8%) | 35/275 (12.7%) | 5/1374 (0.4%) |  |  |  |  |  |  |  |
| Weeks since second vaccination: Median (IQR) | -1.0 (-4.0, 2.0) | 32.0 (28.0, 34.0) |  |  | 3.0 (2.0, 5.0) | 25.0 (20.0, 28.0) | 33.0 (31.0, 35.0) |  |  |  | 4.0 (2.0, 6.0) |
| Second vaccination received: AZD1222, n (%) | 1858/3266 (56.9%) | 1888/3275 (57.6%) |  |  | 212/748 (28.3%) | 411/691 (59.5%) | 1065/1934 (55.1%) |  |  |  | 50/284 (17.6%) |
| Second vaccination received: BNT162b2, n (%) | 1357/3266 (41.5%) | 1330/3275 (40.6%) |  |  | 532/748 (71.1%) | 241/691 (34.9%) | 858/1934 (44.4%) |  |  |  | 234/284 (82.4%) |
| Second vaccination received: Other, n (%) | 51/3266 (1.6%) | 57/3275 (1.7%) |  |  | < 5 | 39/691 (5.6%) | 11/1934 (0.6%) |  |  |  |  |
| Weeks since third vaccination: Median (IQR) | -28.0 (-30.0, -26.0) | 5.0 (3.0, 7.0) |  |  |  |  | 5.0 (4.0, 8.0) |  |  |  |  |
| Third vaccination received: mRNA-1273, n (%) | 293/2149 (13.6%) | 337/2400 (14.0%) |  |  |  |  | 203/1903 (10.7%) |  |  |  |  |
| Third vaccination received: BNT162b2, n (%) | 1828/2149 (85.1%) | 2026/2400 (84.4%) |  |  |  |  | 1677/1903 (88.1%) |  |  |  |  |
| Third vaccination received: Other, n (%) | 28/2149 (1.3%) | 37/2400 (1.5%) |  |  |  |  | 23/1903 (1.2%) |  |  |  |  |
| SARS-CoV-2 infection status (serology-based) at time of antibody testing: Evidence of natural infection, n (%) | 891/4190 (21.3%) | 977/3561 (27.4%) | 98/330 (29.7%) | 304/1375 (22.1%) | 157/748 (21.0%) | 245/691 (35.5%) | 464/1937 (24.0%) | 187/1757 (10.6%) | 23/36 (63.9%) | 133/1438 (9.2%) | 31/283 (11.0%) |
| SARS-CoV-2 infection status (self-reported), Q2: Unsure, n (%) | 93/4092 (2.3%) | 60/3428 (1.8%) | 11/320 (3.4%) | 43/1365 (3.2%) | 9/739 (1.2%) | 12/662 (1.8%) | 30/1882 (1.6%) |  |  |  |  |
| SARS-CoV-2 infection status (self-reported), Q2: Suspected case, n (%) | 477/4092 (11.7%) | 399/3428 (11.6%) | 35/320 (10.9%) | 183/1365 (13.4%) | 67/739 (9.1%) | 81/662 (12.2%) | 197/1882 (10.5%) | 302/1675 (18.0%) | 5/33 (15.2%) | 240/1374 (17.5%) | 57/268 (21.3%) |
| SARS-CoV-2 infection status (self-reported), Q2: Confirmed case, n (%) | 597/4092 (14.6%) | 492/3428 (14.4%) | 57/320 (17.8%) | 218/1365 (16.0%) | 112/739 (15.2%) | 107/662 (16.2%) | 256/1882 (13.6%) | 40/1675 (2.4%) | < 5 | 29/1374 (2.1%) | 11/268 (4.1%) |
| SARS-CoV-2 infection status (self-reported), Q4: Unsure, n (%) | 147/4134 (3.6%) | 128/3543 (3.6%) | 19/330 (5.8%) | 67/1375 (4.9%) | 18/748 (2.4%) | 30/691 (4.3%) | 65/1936 (3.4%) |  |  |  |  |
| SARS-CoV-2 infection status (self-reported), Q4: Suspected case, n (%) | 478/4134 (11.6%) | 404/3543 (11.4%) | 34/330 (10.3%) | 183/1375 (13.3%) | 70/748 (9.4%) | 78/691 (11.3%) | 204/1936 (10.5%) |  |  |  |  |
| SARS-CoV-2 infection status (self-reported), Q4: Confirmed case, n (%) | 817/4134 (19.8%) | 751/3543 (21.2%) | 92/330 (27.9%) | 306/1375 (22.3%) | 145/748 (19.4%) | 202/691 (29.2%) | 357/1936 (18.4%) |  |  |  |  |
| Anti-Nucleocapsid antibody status, Q2: Positive, n (%) | 460/3893 (11.8%) | 333/2887 (11.5%) | 60/329 (18.2%) | 156/1368 (11.4%) | 87/743 (11.7%) | 85/565 (15.0%) | 160/1624 (9.9%) | 167/1757 (9.5%) | < 5 | 133/1438 (9.2%) | 31/283 (11.0%) |
| Anti-Nucleocapsid antibody status, Q4: Positive, n (%) | 524/2998 (17.5%) | 618/3447 (17.9%) | 80/290 (27.6%) | 197/1130 (17.4%) | 95/602 (15.8%) | 179/691 (25.9%) | 263/1937 (13.6%) |  |  |  |  |
| Frailty Index: Frail, n (%) | 390/3316 (11.8%) | 312/2854 (10.9%) | 9/195 (4.6%) | 111/1138 (9.8%) | 90/622 (14.5%) | 45/478 (9.4%) | 192/1665 (11.5%) |  |  |  |  |
| Frailty Index: Very frail, n (%) | 112/3316 (3.4%) | 91/2854 (3.2%) | < 5 | 35/1138 (3.1%) | 23/622 (3.7%) | 7/478 (1.5%) | 56/1665 (3.4%) |  |  |  |  |
| Frailty (PRISMA-7): Frail, n (%) |  |  |  |  |  |  |  | 50/1779 (2.8%) | < 5 | 40/1459 (2.7%) | 10/284 (3.5%) |
| Advised on "Shielded Patient List": Yes, n (%) | 341/4109 (8.3%) | 279/3530 (7.9%) | 8/329 (2.4%) | 82/1374 (6.0%) | 86/748 (11.5%) | 23/691 (3.3%) | 190/1936 (9.8%) | 67/1754 (3.8%) | < 5 | 45/1443 (3.1%) | 22/276 (8.0%) |
| Prescribed immunosuppressant medication: Yes, n (%) | 345/2824 (12.2%) | 291/2444 (11.9%) | 9/169 (5.3%) | 117/963 (12.1%) | 64/540 (11.9%) | 37/394 (9.4%) | 192/1462 (13.1%) |  |  |  |  |
| Immunocompromised: Yes, n (%) |  |  |  |  |  |  |  | 54/1332 (4.1%) | < 5 | 39/1092 (3.6%) | 15/213 (7.0%) |
| Self-rated health: Poor, Fair, n (%) | 357/4082 (8.7%) | 290/3407 (8.5%) | 15/316 (4.7%) | 134/1364 (9.8%) | 60/737 (8.1%) | 42/656 (6.4%) | 168/1871 (9.0%) | 167/1778 (9.4%) | < 5 | 137/1459 (9.4%) | 28/283 (9.9%) |
| BMI: Median (IQR) | 24.75 (22.15, 27.99) | 24.76 (22.2, 27.98) | 22.72 (20.94, 25.42) | 24.86 (22.27, 28.28) | 24.99 (22.23, 28.07) | 23.91 (21.62, 27.58) | 24.87 (22.3, 27.87) | 25.7 (23.23, 28.7) | 25.63 (23.13, 28.04) | 25.71 (23.25, 28.77) | 25.65 (23.02, 28.6) |
| Number of selected comorbidities: 1+, n (%) | 1345/3047 (44.1%) | 1134/2617 (43.3%) | 46/190 (24.2%) | 452/1056 (42.8%) | 288/555 (51.9%) | 144/452 (31.9%) | 717/1506 (47.6%) | 387/1289 (30.0%) | 6/26 (23.1%) | 314/1058 (29.7%) | 67/205 (32.7%) |
| Comorbidity: Anxiety or Stress Disorder: Yes, n (%) | 474/3071 (15.4%) | 389/2635 (14.8%) | 29/177 (16.4%) | 165/1059 (15.6%) | 81/576 (14.1%) | 70/441 (15.9%) | 213/1548 (13.8%) | 131/1321 (9.9%) | < 5 | 114/1083 (10.5%) | 16/211 (7.6%) |
| Comorbidity: Depression: Yes, n (%) | 387/3039 (12.7%) | 328/2618 (12.5%) | 22/174 (12.6%) | 134/1051 (12.7%) | 70/574 (12.2%) | 54/437 (12.4%) | 180/1533 (11.7%) | 65/1330 (4.9%) | < 5 | 55/1092 (5.0%) | 9/211 (4.3%) |
| Anxiety (HADS) score up to Q2: Median (IQR) | 6.0 (3.0, 9.0) | 6.0 (3.0, 9.0) | 7.0 (4.0, 11.0) | 6.0 (3.0, 10.0) | 6.0 (3.0, 9.0) | 7.0 (4.0, 10.0) | 6.0 (3.0, 9.0) |  |  |  |  |
| Anxiety (HADS) score up to Q2: 8-10, mild, n (%) | 874/4074 (21.5%) | 730/3420 (21.3%) | 75/319 (23.5%) | 298/1361 (21.9%) | 153/736 (20.8%) | 143/661 (21.6%) | 392/1878 (20.9%) |  |  |  |  |
| Anxiety (HADS) score up to Q2: 11+, moderate, severe, n (%) | 752/4074 (18.5%) | 601/3420 (17.6%) | 82/319 (25.7%) | 262/1361 (19.3%) | 120/736 (16.3%) | 137/661 (20.7%) | 276/1878 (14.7%) |  |  |  |  |
| Depression (HADS) score up to Q2: Median (IQR) | 5.0 (2.0, 8.0) | 5.0 (2.0, 7.0) | 5.0 (3.0, 8.0) | 5.0 (2.0, 8.0) | 4.0 (2.0, 7.0) | 5.0 (3.0, 8.0) | 4.0 (2.0, 7.0) |  |  |  |  |
| Depression (HADS) score up to Q2: 8-10, mild, n (%) | 627/4076 (15.4%) | 511/3419 (14.9%) | 54/319 (16.9%) | 227/1362 (16.7%) | 112/736 (15.2%) | 95/660 (14.4%) | 275/1878 (14.6%) |  |  |  |  |
| Depression (HADS) score up to Q2: 11+, moderate, severe, n (%) | 398/4076 (9.8%) | 331/3419 (9.7%) | 36/319 (11.3%) | 151/1362 (11.1%) | 60/736 (8.2%) | 85/660 (12.9%) | 146/1878 (7.8%) |  |  |  |  |
| Anxiety (HADS) score up to Q4: Median (IQR) | 7.0 (4.0, 10.0) | 7.0 (4.0, 10.0) | 8.0 (5.0, 11.0) | 7.0 (4.0, 10.0) | 6.0 (3.0, 9.0) | 7.0 (5.0, 10.5) | 6.0 (3.0, 9.0) |  |  |  |  |
| Anxiety (HADS) score up to Q4: 8-10, mild, n (%) | 900/4121 (21.8%) | 776/3541 (21.9%) | 75/329 (22.8%) | 309/1372 (22.5%) | 160/746 (21.4%) | 144/691 (20.8%) | 431/1937 (22.3%) |  |  |  |  |
| Anxiety (HADS) score up to Q4: 11+, moderate, severe, n (%) | 846/4121 (20.5%) | 715/3541 (20.2%) | 98/329 (29.8%) | 297/1372 (21.6%) | 138/746 (18.5%) | 173/691 (25.0%) | 327/1937 (16.9%) |  |  |  |  |
| Depression (HADS) score up to Q4: Median (IQR) | 5.0 (3.0, 8.0) | 5.0 (3.0, 8.0) | 6.0 (3.0, 8.0) | 5.0 (3.0, 8.0) | 5.0 (3.0, 8.0) | 6.0 (3.0, 8.0) | 5.0 (3.0, 8.0) |  |  |  |  |
| Depression (HADS) score up to Q4: 8-10, mild, n (%) | 693/4124 (16.8%) | 598/3541 (16.9%) | 60/329 (18.2%) | 244/1373 (17.8%) | 129/746 (17.3%) | 106/691 (15.3%) | 331/1937 (17.1%) |  |  |  |  |
| Depression (HADS) score up to Q4: 11+, moderate, severe, n (%) | 455/4124 (11.0%) | 395/3541 (11.2%) | 46/329 (14.0%) | 175/1373 (12.7%) | 67/746 (9.0%) | 105/691 (15.2%) | 174/1937 (9.0%) |  |  |  |  |
