# Supplementary material for: Antibody levels following vaccination against SARS-CoV-2: associations with post-vaccination infection and risk factors in two UK longitudinal studies
Source: eLife. 2023 Jan 24;12:e80428. doi: 10.7554/eLife.80428 (PMC9940912; doi:10.7554/eLife.80428)
Supplement: Supplementary file 3. — p-Values are generated from chi-square test of independence on cross tabulation of counts for the socio-demographic variable of interest and all categories (including those not presented) of the SARS-CoV-2 infection variable. [file elife-80428-supp3.docx]

Supplementary file 3. SARS-CoV-2 infection prevalence rates, split by selected socio-demographic variables, for TwinsUK Q4 antibody testing participants. P-values are generated from chi-square test of independence on cross tabulation of counts for the socio-demographic variable of interest and all categories (including those not presented) of the SARS-CoV-2 infection variable.

| **Variable** | **SARS-CoV-2 infection status (self-reported): Suspected case** | **SARS-CoV-2 infection status (self-reported): Confirmed case** | **SARS-CoV-2 infection status (serology-based): Evidence of natural infection** | **Anti-Nucleocapsid antibody status: Positive** |
| --- | --- | --- | --- | --- |
| Overall | 404/3543 (11.4%) | 751/3543 (21.2%) | 977/3560 (27.4%) | 618/3447 (17.9%) |
| Sex: Female | 362/3101 (11.7%) [p = 0.41] | 650/3101 (21.0%) [p = 0.41] | 837/3113 (26.9%) [p = 0.03] | 530/3016 (17.6%) [p = 0.18] |
| Sex: Male | 41/441 (9.3%) | 101/441 (22.9%) | 139/446 (31.2%) | 87/430 (20.2%) |
| Age: 18-29 | 14/131 (10.7%) [p < 0.0001] | 44/131 (33.6%) [p < 0.0001] | 60/133 (45.1%) [p < 0.0001] | 48/130 (36.9%) [p < 0.0001] |
| Age: 30-39 | 43/292 (14.7%) | 93/292 (31.8%) | 116/298 (38.9%) | 80/290 (27.6%) |
| Age: 40-49 | 57/376 (15.2%) | 107/376 (28.5%) | 141/380 (37.1%) | 107/374 (28.6%) |
| Age: 50-59 | 94/671 (14.0%) | 183/671 (27.3%) | 224/675 (33.2%) | 168/658 (25.5%) |
| Age: 60-69 | 109/904 (12.1%) | 175/904 (19.4%) | 228/905 (25.2%) | 140/884 (15.8%) |
| Age: 70-79 | 74/962 (7.7%) | 130/962 (13.5%) | 174/964 (18.0%) | 67/928 (7.2%) |
| Age: 80+ | 13/207 (6.3%) | 19/207 (9.2%) | 34/205 (16.6%) | 8/183 (4.4%) |
| Ethnicity: Other than white | 12/96 (12.5%) [p = 0.74] | 22/96 (22.9%) [p = 0.74] | 44/96 (45.8%) [p = 0.0001] | 21/96 (21.9%) [p = 0.30] |
| Ethnicity: White | 385/3411 (11.3%) | 724/3411 (21.2%) | 925/3426 (27.0%) | 589/3314 (17.8%) |
| IMD: Quintile 1 (most deprived 20%) | 29/206 (14.1%) [p = 0.002] | 60/206 (29.1%) [p = 0.002] | 71/210 (33.8%) [p = 0.21] | 56/200 (28.0%) [p = 0.004] |
| IMD: Quintile 2 | 54/423 (12.8%) | 77/423 (18.2%) | 120/426 (28.2%) | 77/409 (18.8%) |
| IMD: Quintile 3 | 95/725 (13.1%) | 147/725 (20.3%) | 189/724 (26.1%) | 116/697 (16.6%) |
| IMD: Quintile 4 | 118/918 (12.9%) | 185/918 (20.2%) | 244/927 (26.3%) | 153/900 (17.0%) |
| IMD: Quintile 5 (least deprived 20%) | 107/1261 (8.5%) | 281/1261 (22.3%) | 351/1262 (27.8%) | 215/1230 (17.5%) |
| RUC: Rural | 106/911 (11.6%) [p = 0.07] | 168/911 (18.4%) [p = 0.07] | 203/912 (22.3%) [p < 0.0001] | 125/884 (14.1%) [p = 0.0005] |
| RUC: Urban | 296/2603 (11.4%) | 581/2603 (22.3%) | 770/2617 (29.4%) | 490/2532 (19.4%) |
