# Supplementary material for: Antibody levels following vaccination against SARS-CoV-2: associations with post-vaccination infection and risk factors in two UK longitudinal studies
Source: eLife. 2023 Jan 24;12:e80428. doi: 10.7554/eLife.80428 (PMC9940912; doi:10.7554/eLife.80428)
Supplement: Supplementary file 4. — The antibody level assay range is 0.4–250 BAU/mL for Q2 results and 0.4–25000 BAU/mL for Q4 results, with a positive threshold of 0.8 BAU/mL. [file elife-80428-supp4.docx]

Supplementary file 4. Anti-Spike antibody levels and weeks since most recent vaccination within TwinsUK and ALSPAC individuals, stratified by vaccination status at Q2 and Q4 antibody testing, split by various variables. The antibody level assay range is 0.4 to 250 BAU/mL for Q2 results and 0.4 to 25,000 BAU/mL for Q4 results, with a positive threshold of 0.8 BAU/mL.

| **Cohort** | **TwinsUK** | | | | **ALSPAC** | |
| --- | --- | --- | --- | --- | --- | --- |
| **Testing period** | **Q2** | | **Q4** | | **Q2** | |
| **Vaccination status** | **Single-vaccinated** | **Double-vaccinated** | **Double-vaccinated** | **Triple-vaccinated** | **Single-vaccinated** | **Double-vaccinated** |
| Age group: 18-49, Anti-Spike level | n = 145, Median: 52.58, IQR: (19.25, 180.6), 5%: 4.57, 10%: 8.22 | n = 123, Median: 250.0, IQR: (250.0, 250.0), 5%: 250.0, 10%: 250.0 | n = 352, Median: 2130.5, IQR: (485.0, 5662.75), 5%: 91.76, 10%: 201.5 | n = 215, Median: 12844.0, IQR: (8257.0, 22363.5), 5%: 3310.8, 10%: 4771.0 | n = 8, Median: 167.77, IQR: (57.98, 250.0), 5%: 21.52, 10%: 25.04 | n = 6, Median: 250.0, IQR: (250.0, 250.0), 5%: 250.0, 10%: 250.0 |
| Age group: 18-49, Weeks since vaccination | Median: 6.0, IQR: (5.0, 8.0) | Median: 5.0, IQR: (3.0, 6.5) | Median: 21.0, IQR: (18.0, 24.0) | Median: 6.0, IQR: (4.0, 9.0) | Median: 6.0, IQR: (5.0, 7.0) | Median: 6.0, IQR: (2.75, 12.25) |
| Age group: 50-69, Anti-Spike level | n = 944, Median: 52.97, IQR: (20.84, 124.9), 5%: 4.01, 10%: 8.73 | n = 249, Median: 250.0, IQR: (250.0, 250.0), 5%: 146.86, 10%: 250.0 | n = 293, Median: 701.0, IQR: (236.0, 3692.0), 5%: 66.94, 10%: 87.28 | n = 815, Median: 15607.0, IQR: (9223.0, 25000.0), 5%: 3946.2, 10%: 5342.6 | n = 1445, Median: 43.28, IQR: (17.82, 105.1), 5%: 3.2, 10%: 6.49 | n = 268, Median: 250.0, IQR: (250.0, 250.0), 5%: 156.69, 10%: 250.0 |
| Age group: 50-69, Weeks since vaccination | Median: 7.0, IQR: (5.0, 9.0) | Median: 4.0, IQR: (2.0, 6.0) | Median: 27.0, IQR: (25.0, 29.0) | Median: 5.0, IQR: (3.0, 7.0) | Median: 6.0, IQR: (5.0, 8.0) | Median: 4.0, IQR: (3.0, 6.0) |
| Age group: 70+, Anti-Spike level | n = 286, Median: 56.87, IQR: (27.99, 108.88), 5%: 7.2, 10%: 13.55 | n = 376, Median: 250.0, IQR: (250.0, 250.0), 5%: 132.8, 10%: 250.0 | n = 46, Median: 3807.5, IQR: (456.25, 12092.5), 5%: 83.45, 10%: 111.15 | n = 907, Median: 12569.0, IQR: (7354.0, 21218.5), 5%: 3128.9, 10%: 4835.0 | n = 6, Median: 72.51, IQR: (18.69, 120.88), 5%: 13.82, 10%: 14.3 | n = 10, Median: 250.0, IQR: (250.0, 250.0), 5%: 124.86, 10%: 227.25 |
| Age group: 70+, Weeks since vaccination | Median: 10.0, IQR: (9.0, 10.0) | Median: 3.0, IQR: (2.0, 4.0) | Median: 31.5, IQR: (29.0, 33.0) | Median: 6.0, IQR: (4.0, 8.0) | Median: 9.0, IQR: (9.0, 9.75) | Median: 2.5, IQR: (2.0, 3.75) |
| Sex: Female, Anti-Spike level | n = 1197, Median: 57.03, IQR: (24.64, 124.9), 5%: 4.78, 10%: 9.88 | n = 660, Median: 250.0, IQR: (250.0, 250.0), 5%: 147.24, 10%: 250.0 | n = 588, Median: 1162.0, IQR: (321.0, 5011.5), 5%: 73.9, 10%: 110.8 | n = 1712, Median: 13803.0, IQR: (8201.5, 23702.75), 5%: 3438.6, 10%: 5027.1 | n = 1062, Median: 45.15, IQR: (19.2, 108.02), 5%: 3.37, 10%: 7.37 | n = 238, Median: 250.0, IQR: (250.0, 250.0), 5%: 245.0, 10%: 250.0 |
| Sex: Female, Weeks since vaccination | Median: 8.0, IQR: (6.0, 9.0) | Median: 3.0, IQR: (2.0, 5.0) | Median: 25.0, IQR: (20.0, 28.0) | Median: 5.0, IQR: (4.0, 8.0) | Median: 6.0, IQR: (5.0, 8.0) | Median: 4.0, IQR: (2.0, 6.0) |
| Sex: Male, Anti-Spike level | n = 178, Median: 38.9, IQR: (15.61, 99.0), 5%: 3.39, 10%: 7.34 | n = 88, Median: 250.0, IQR: (250.0, 250.0), 5%: 196.51, 10%: 250.0 | n = 103, Median: 2349.0, IQR: (410.0, 5971.0), 5%: 66.84, 10%: 132.4 | n = 225, Median: 13220.0, IQR: (7794.0, 21949.0), 5%: 4085.6, 10%: 5325.8 | n = 397, Median: 39.47, IQR: (16.34, 98.81), 5%: 2.82, 10%: 5.93 | n = 46, Median: 250.0, IQR: (250.0, 250.0), 5%: 34.21, 10%: 110.76 |
| Sex: Male, Weeks since vaccination | Median: 8.0, IQR: (6.0, 9.0) | Median: 3.0, IQR: (2.0, 6.0) | Median: 25.0, IQR: (18.0, 28.0) | Median: 6.0, IQR: (4.0, 8.0) | Median: 7.0, IQR: (5.0, 8.0) | Median: 4.0, IQR: (2.25, 6.75) |
| Ethnicity: White, Anti-Spike level | n = 1339, Median: 53.18, IQR: (22.72, 121.2), 5%: 4.72, 10%: 9.54 | n = 720, Median: 250.0, IQR: (250.0, 250.0), 5%: 147.65, 10%: 250.0 | n = 660, Median: 1272.0, IQR: (324.25, 5262.5), 5%: 73.5, 10%: 108.0 | n = 1875, Median: 13704.0, IQR: (8199.0, 23491.5), 5%: 3497.6, 10%: 5058.0 | n = 1434, Median: 42.82, IQR: (17.81, 104.4), 5%: 3.2, 10%: 6.49 | n = 279, Median: 250.0, IQR: (250.0, 250.0), 5%: 159.66, 10%: 250.0 |
| Ethnicity: White, Weeks since vaccination | Median: 8.0, IQR: (6.0, 9.0) | Median: 3.0, IQR: (2.0, 5.0) | Median: 25.0, IQR: (20.0, 28.0) | Median: 5.0, IQR: (4.0, 8.0) | Median: 6.0, IQR: (5.0, 8.0) | Median: 4.0, IQR: (2.0, 6.0) |
| Ethnicity: Other than White, Anti-Spike level | n = 29, Median: 70.85, IQR: (43.27, 138.8), 5%: 7.61, 10%: 18.61 | n = 19, Median: 250.0, IQR: (250.0, 250.0), 5%: 250.0, 10%: 250.0 | n = 26, Median: 2576.5, IQR: (880.5, 4011.5), 5%: 332.75, 10%: 503.5 | n = 39, Median: 13992.0, IQR: (7064.0, 25000.0), 5%: 3138.3, 10%: 4557.8 | n = 20, Median: 104.45, IQR: (38.22, 250.0), 5%: 12.8, 10%: 15.96 | n = 5, Median: 250.0, IQR: (250.0, 250.0), 5%: 105.53, 10%: 141.65 |
| Ethnicity: Other than White, Weeks since vaccination | Median: 6.0, IQR: (5.0, 8.0) | Median: 4.0, IQR: (3.0, 6.0) | Median: 22.5, IQR: (19.0, 28.0) | Median: 6.0, IQR: (3.5, 9.0) | Median: 7.0, IQR: (5.0, 8.25) | Median: 7.0, IQR: (5.0, 7.0) |
| Local area deprivation, IMD: Least deprived 60% (decile 5-10), Anti-Spike level | n = 1157, Median: 52.32, IQR: (23.04, 119.2), 5%: 5.0, 10%: 10.0 | n = 624, Median: 250.0, IQR: (250.0, 250.0), 5%: 161.07, 10%: 250.0 | n = 539, Median: 1251.0, IQR: (323.5, 5132.5), 5%: 73.85, 10%: 108.0 | n = 1635, Median: 13728.0, IQR: (8248.5, 23222.0), 5%: 3543.4, 10%: 5058.4 | n = 666, Median: 44.08, IQR: (19.06, 110.4), 5%: 3.05, 10%: 6.54 | n = 148, Median: 250.0, IQR: (250.0, 250.0), 5%: 238.0, 10%: 250.0 |
| Local area deprivation, IMD: Least deprived 60% (decile 5-10), Weeks since vaccination | Median: 8.0, IQR: (6.0, 9.0) | Median: 3.0, IQR: (2.0, 5.0) | Median: 25.0, IQR: (20.0, 28.0) | Median: 5.0, IQR: (4.0, 8.0) | Median: 6.0, IQR: (5.0, 7.0) | Median: 4.0, IQR: (3.0, 6.0) |
| Local area deprivation, IMD: Most deprived 40% (decile 1-4), Anti-Spike level | n = 214, Median: 58.87, IQR: (20.36, 147.02), 5%: 3.58, 10%: 6.52 | n = 124, Median: 250.0, IQR: (250.0, 250.0), 5%: 95.82, 10%: 250.0 | n = 151, Median: 1616.0, IQR: (399.5, 5481.0), 5%: 73.7, 10%: 127.0 | n = 298, Median: 13542.5, IQR: (7234.5, 25000.0), 5%: 3058.25, 10%: 4825.6 | n = 305, Median: 45.21, IQR: (18.97, 107.1), 5%: 3.42, 10%: 7.4 | n = 70, Median: 250.0, IQR: (250.0, 250.0), 5%: 250.0, 10%: 250.0 |
| Local area deprivation, IMD: Most deprived 40% (decile 1-4), Weeks since vaccination | Median: 8.0, IQR: (6.0, 9.0) | Median: 3.0, IQR: (2.0, 5.0) | Median: 25.0, IQR: (19.5, 29.0) | Median: 5.0, IQR: (3.0, 8.0) | Median: 6.0, IQR: (5.0, 7.0) | Median: 3.0, IQR: (2.0, 6.0) |
| Highest educational attainment: NVQ level 3 or lower, Anti-Spike level | n = 497, Median: 55.56, IQR: (24.64, 119.8), 5%: 5.39, 10%: 10.8 | n = 275, Median: 250.0, IQR: (250.0, 250.0), 5%: 62.1, 10%: 181.34 | n = 171, Median: 951.0, IQR: (296.0, 5211.5), 5%: 64.05, 10%: 100.0 | n = 763, Median: 13768.0, IQR: (8100.5, 23719.5), 5%: 3388.4, 10%: 4898.0 | n = 936, Median: 45.78, IQR: (17.99, 115.85), 5%: 2.76, 10%: 5.62 | n = 210, Median: 250.0, IQR: (250.0, 250.0), 5%: 163.08, 10%: 250.0 |
| Highest educational attainment: NVQ level 3 or lower, Weeks since vaccination | Median: 8.0, IQR: (6.0, 10.0) | Median: 3.0, IQR: (2.0, 5.0) | Median: 27.0, IQR: (24.5, 30.0) | Median: 5.0, IQR: (4.0, 8.0) | Median: 6.0, IQR: (5.0, 8.0) | Median: 4.0, IQR: (3.0, 6.0) |
| Highest educational attainment: NVQ level 4 or higher, Anti-Spike level | n = 709, Median: 52.58, IQR: (22.76, 117.8), 5%: 4.57, 10%: 9.63 | n = 377, Median: 250.0, IQR: (250.0, 250.0), 5%: 226.0, 10%: 250.0 | n = 354, Median: 1485.0, IQR: (353.5, 5717.5), 5%: 73.76, 10%: 106.6 | n = 969, Median: 13589.0, IQR: (8089.0, 23543.0), 5%: 3570.2, 10%: 5072.0 | n = 452, Median: 39.61, IQR: (18.4, 94.4), 5%: 5.08, 10%: 8.56 | n = 60, Median: 250.0, IQR: (250.0, 250.0), 5%: 148.9, 10%: 244.08 |
| Highest educational attainment: NVQ level 4 or higher, Weeks since vaccination | Median: 8.0, IQR: (6.0, 9.0) | Median: 3.0, IQR: (2.0, 5.0) | Median: 25.0, IQR: (20.0, 28.0) | Median: 5.0, IQR: (3.0, 8.0) | Median: 7.0, IQR: (5.0, 8.0) | Median: 4.0, IQR: (2.0, 7.0) |
| First vaccination received: AZD1222, Anti-Spike level | n = 1103, Median: 48.45, IQR: (19.8, 116.05), 5%: 3.89, 10%: 7.89 |  |  |  | n = 1235, Median: 39.26, IQR: (16.42, 103.45), 5%: 2.82, 10%: 5.8 | n = 50, Median: 250.0, IQR: (163.6, 250.0), 5%: 22.95, 10%: 79.62 |
| First vaccination received: AZD1222, Weeks since vaccination | Median: 7.0, IQR: (5.0, 9.0) |  |  |  | Median: 6.0, IQR: (5.0, 7.0) | Median: 3.0, IQR: (2.0, 4.0) |
| First vaccination received: BNT162b2, Anti-Spike level | n = 266, Median: 74.33, IQR: (35.96, 145.3), 5%: 13.41, 10%: 19.29 |  |  |  | n = 224, Median: 59.66, IQR: (31.04, 118.18), 5%: 9.29, 10%: 16.14 | n = 234, Median: 250.0, IQR: (250.0, 250.0), 5%: 250.0, 10%: 250.0 |
| First vaccination received: BNT162b2, Weeks since vaccination | Median: 9.0, IQR: (8.0, 10.0) |  |  |  | Median: 8.0, IQR: (7.0, 9.0) | Median: 4.0, IQR: (3.0, 7.0) |
| First vaccination received: Other, Anti-Spike level | n = 5, Median: 57.86, IQR: (42.05, 107.0), 5%: 39.03, 10%: 39.79 |  |  |  |  |  |
| First vaccination received: Other, Weeks since vaccination | Median: 10.0, IQR: (8.0, 10.0) |  |  |  |  |  |
| Second vaccination received: AZD1222, Anti-Spike level |  | n = 212, Median: 250.0, IQR: (250.0, 250.0), 5%: 91.47, 10%: 171.93 | n = 411, Median: 459.0, IQR: (216.5, 2721.0), 5%: 60.45, 10%: 83.4 | n = 1065, Median: 14358.0, IQR: (8523.0, 23950.0), 5%: 3636.4, 10%: 5260.2 |  |  |
| Second vaccination received: AZD1222, Weeks since vaccination |  | Median: 3.0, IQR: (2.0, 3.25) | Median: 26.0, IQR: (24.0, 28.0) | Median: 5.0, IQR: (3.0, 7.0) |  |  |
| Second vaccination received: BNT162b2, Anti-Spike level |  | n = 532, Median: 250.0, IQR: (250.0, 250.0), 5%: 250.0, 10%: 250.0 | n = 241, Median: 3174.0, IQR: (1424.0, 6406.0), 5%: 571.0, 10%: 731.0 | n = 858, Median: 13118.0, IQR: (7619.25, 22570.5), 5%: 3312.9, 10%: 4782.6 |  |  |
| Second vaccination received: BNT162b2, Weeks since vaccination |  | Median: 4.0, IQR: (2.75, 6.0) | Median: 20.0, IQR: (17.0, 30.0) | Median: 7.0, IQR: (4.0, 9.0) |  |  |
| Second vaccination received: Other, Anti-Spike level |  | n < 5, Median: 250.0, IQR: (229.42, 250.0), 5%: 180.04, 10%: 192.39 | n = 39, Median: 4222.0, IQR: (3107.0, 7033.0), 5%: 1382.9, 10%: 2231.2 | n = 11, Median: 14195.0, IQR: (10306.5, 21298.0), 5%: 7422.0, 10%: 8391.0 |  |  |
| Second vaccination received: Other, Weeks since vaccination |  | Median: 8.0, IQR: (5.5, 10.0) | Median: 19.0, IQR: (16.0, 21.5) | Median: 5.0, IQR: (4.0, 7.5) |  |  |
| Third vaccination received: mRNA-1273, Anti-Spike level |  |  |  | n = 203, Median: 22154.0, IQR: (14025.0, 25000.0), 5%: 6129.1, 10%: 8709.4 |  |  |
| Third vaccination received: mRNA-1273, Weeks since vaccination |  |  |  | Median: 3.0, IQR: (2.0, 5.0) |  |  |
| Third vaccination received: BNT162b2, Anti-Spike level |  |  |  | n = 1677, Median: 13170.0, IQR: (7743.0, 22100.0), 5%: 3262.0, 10%: 4865.4 |  |  |
| Third vaccination received: BNT162b2, Weeks since vaccination |  |  |  | Median: 6.0, IQR: (4.0, 8.0) |  |  |
| Third vaccination received: Other, Anti-Spike level |  |  |  | n = 23, Median: 16475.0, IQR: (8934.0, 23189.5), 5%: 1489.5, 10%: 6515.6 |  |  |
| Third vaccination received: Other, Weeks since vaccination |  |  |  | Median: 5.0, IQR: (4.0, 7.5) |  |  |
| SARS-CoV-2 infection status (serology-based) at time of antibody testing: No evidence of natural infection, Anti-Spike level | n = 1071, Median: 43.94, IQR: (19.72, 87.77), 5%: 4.04, 10%: 8.7 | n = 591, Median: 250.0, IQR: (250.0, 250.0), 5%: 136.35, 10%: 250.0 | n = 444, Median: 603.5, IQR: (237.75, 2460.0), 5%: 61.08, 10%: 84.41 | n = 1471, Median: 12883.0, IQR: (7551.0, 21130.5), 5%: 3227.0, 10%: 4828.0 | n = 1305, Median: 38.2, IQR: (16.29, 83.52), 5%: 2.93, 10%: 6.07 | n = 252, Median: 250.0, IQR: (250.0, 250.0), 5%: 152.24, 10%: 250.0 |
| SARS-CoV-2 infection status (serology-based) at time of antibody testing: No evidence of natural infection, Weeks since vaccination | Median: 8.0, IQR: (6.0, 9.0) | Median: 3.0, IQR: (2.0, 5.0) | Median: 25.0, IQR: (20.0, 28.0) | Median: 5.0, IQR: (4.0, 8.0) | Median: 6.0, IQR: (5.0, 8.0) | Median: 4.0, IQR: (2.0, 6.0) |
| SARS-CoV-2 infection status (serology-based) at time of antibody testing: Evidence of natural infection, Anti-Spike level | n = 304, Median: 250.0, IQR: (52.84, 250.0), 5%: 7.68, 10%: 18.84 | n = 157, Median: 250.0, IQR: (250.0, 250.0), 5%: 250.0, 10%: 250.0 | n = 245, Median: 5051.0, IQR: (1750.0, 13098.0), 5%: 247.4, 10%: 465.8 | n = 464, Median: 18238.0, IQR: (10392.25, 25000.0), 5%: 4690.5, 10%: 6661.0 | n = 133, Median: 250.0, IQR: (250.0, 250.0), 5%: 155.18, 10%: 250.0 | n = 31, Median: 250.0, IQR: (250.0, 250.0), 5%: 250.0, 10%: 250.0 |
| SARS-CoV-2 infection status (serology-based) at time of antibody testing: Evidence of natural infection, Weeks since vaccination | Median: 8.0, IQR: (6.0, 9.0) | Median: 3.0, IQR: (2.0, 5.0) | Median: 25.0, IQR: (20.0, 28.0) | Median: 5.0, IQR: (3.0, 8.0) | Median: 6.0, IQR: (5.0, 8.0) | Median: 4.0, IQR: (3.0, 6.0) |
| SARS-CoV-2 infection status (self-reported), Q2: No infection, Anti-Spike level | n = 921, Median: 46.19, IQR: (20.35, 92.63), 5%: 3.69, 10%: 8.42 | n = 551, Median: 250.0, IQR: (250.0, 250.0), 5%: 119.45, 10%: 250.0 | n = 462, Median: 989.5, IQR: (293.0, 4610.75), 5%: 66.45, 10%: 100.1 | n = 1399, Median: 13408.0, IQR: (7612.0, 23167.5), 5%: 3245.4, 10%: 4823.6 | n = 1105, Median: 40.23, IQR: (17.09, 94.07), 5%: 3.03, 10%: 6.21 | n = 200, Median: 250.0, IQR: (250.0, 250.0), 5%: 154.52, 10%: 250.0 |
| SARS-CoV-2 infection status (self-reported), Q2: No infection, Weeks since vaccination | Median: 8.0, IQR: (6.0, 10.0) | Median: 3.0, IQR: (2.0, 5.0) | Median: 25.0, IQR: (20.0, 28.0) | Median: 6.0, IQR: (4.0, 8.0) | Median: 6.0, IQR: (5.0, 8.0) | Median: 4.0, IQR: (2.0, 6.0) |
| SARS-CoV-2 infection status (self-reported), Q2: Unsure, Anti-Spike level | n = 43, Median: 43.81, IQR: (17.67, 107.7), 5%: 8.96, 10%: 10.85 | n = 9, Median: 250.0, IQR: (250.0, 250.0), 5%: 190.06, 10%: 230.02 | n = 12, Median: 826.5, IQR: (328.75, 3462.0), 5%: 201.15, 10%: 253.3 | n = 30, Median: 20072.5, IQR: (14218.75, 25000.0), 5%: 6379.3, 10%: 7422.6 |  |  |
| SARS-CoV-2 infection status (self-reported), Q2: Unsure, Weeks since vaccination | Median: 8.0, IQR: (5.5, 10.0) | Median: 3.0, IQR: (3.0, 7.0) | Median: 24.0, IQR: (16.75, 25.5) | Median: 3.5, IQR: (3.0, 6.0) |  |  |
| SARS-CoV-2 infection status (self-reported), Q2: Suspected case, Anti-Spike level | n = 183, Median: 50.41, IQR: (20.83, 101.35), 5%: 5.1, 10%: 7.92 | n = 67, Median: 250.0, IQR: (250.0, 250.0), 5%: 239.29, 10%: 250.0 | n = 81, Median: 705.0, IQR: (250.0, 5873.0), 5%: 62.7, 10%: 76.2 | n = 197, Median: 13677.0, IQR: (8263.0, 23933.0), 5%: 3830.0, 10%: 5050.8 | n = 240, Median: 55.47, IQR: (19.76, 153.4), 5%: 3.14, 10%: 7.03 | n = 57, Median: 250.0, IQR: (250.0, 250.0), 5%: 207.89, 10%: 250.0 |
| SARS-CoV-2 infection status (self-reported), Q2: Suspected case, Weeks since vaccination | Median: 8.0, IQR: (5.5, 9.0) | Median: 3.0, IQR: (2.0, 5.0) | Median: 26.0, IQR: (21.0, 29.0) | Median: 6.0, IQR: (4.0, 8.0) | Median: 6.0, IQR: (5.0, 8.0) | Median: 3.0, IQR: (3.0, 7.0) |
| SARS-CoV-2 infection status (self-reported), Q2: Confirmed case, Anti-Spike level | n = 218, Median: 250.0, IQR: (57.28, 250.0), 5%: 11.66, 10%: 23.07 | n = 112, Median: 250.0, IQR: (250.0, 250.0), 5%: 250.0, 10%: 250.0 | n = 107, Median: 3390.0, IQR: (1253.5, 6194.5), 5%: 302.0, 10%: 431.2 | n = 256, Median: 15752.0, IQR: (10174.75, 24951.25), 5%: 5717.75, 10%: 6730.0 | n = 29, Median: 250.0, IQR: (250.0, 250.0), 5%: 107.84, 10%: 250.0 | n = 11, Median: 250.0, IQR: (250.0, 250.0), 5%: 250.0, 10%: 250.0 |
| SARS-CoV-2 infection status (self-reported), Q2: Confirmed case, Weeks since vaccination | Median: 7.0, IQR: (5.0, 9.0) | Median: 4.0, IQR: (3.0, 6.0) | Median: 25.0, IQR: (20.0, 29.0) | Median: 5.0, IQR: (3.0, 8.0) | Median: 5.0, IQR: (5.0, 7.0) | Median: 3.0, IQR: (2.0, 5.0) |
| SARS-CoV-2 infection status (self-reported), Q4: No infection, Anti-Spike level | n = 819, Median: 47.06, IQR: (21.18, 94.3), 5%: 4.06, 10%: 8.88 | n = 515, Median: 250.0, IQR: (250.0, 250.0), 5%: 132.12, 10%: 250.0 | n = 381, Median: 614.0, IQR: (238.0, 2624.0), 5%: 63.6, 10%: 87.1 | n = 1310, Median: 12876.0, IQR: (7374.25, 21687.0), 5%: 3123.9, 10%: 4737.3 |  |  |
| SARS-CoV-2 infection status (self-reported), Q4: No infection, Weeks since vaccination | Median: 8.0, IQR: (6.0, 10.0) | Median: 3.0, IQR: (2.0, 5.0) | Median: 25.0, IQR: (20.0, 28.0) | Median: 6.0, IQR: (4.0, 8.0) |  |  |
| SARS-CoV-2 infection status (self-reported), Q4: Unsure, Anti-Spike level | n = 67, Median: 41.75, IQR: (18.82, 86.56), 5%: 4.61, 10%: 10.17 | n = 18, Median: 250.0, IQR: (250.0, 250.0), 5%: 137.65, 10%: 220.03 | n = 30, Median: 646.5, IQR: (272.75, 3521.0), 5%: 105.81, 10%: 138.2 | n = 65, Median: 17892.0, IQR: (10187.0, 25000.0), 5%: 3924.6, 10%: 6240.4 |  |  |
| SARS-CoV-2 infection status (self-reported), Q4: Unsure, Weeks since vaccination | Median: 8.0, IQR: (5.5, 10.0) | Median: 4.0, IQR: (3.0, 6.75) | Median: 24.5, IQR: (21.0, 27.75) | Median: 4.0, IQR: (3.0, 6.0) |  |  |
| SARS-CoV-2 infection status (self-reported), Q4: Suspected case, Anti-Spike level | n = 183, Median: 48.54, IQR: (19.56, 101.35), 5%: 5.04, 10%: 7.11 | n = 70, Median: 250.0, IQR: (250.0, 250.0), 5%: 241.58, 10%: 250.0 | n = 78, Median: 715.5, IQR: (244.0, 3822.25), 5%: 62.31, 10%: 75.51 | n = 204, Median: 13453.5, IQR: (8192.0, 23315.0), 5%: 3739.0, 10%: 5033.0 |  |  |
| SARS-CoV-2 infection status (self-reported), Q4: Suspected case, Weeks since vaccination | Median: 8.0, IQR: (6.0, 9.0) | Median: 3.0, IQR: (2.0, 5.0) | Median: 25.0, IQR: (20.25, 27.75) | Median: 5.5, IQR: (3.0, 8.0) |  |  |
| SARS-CoV-2 infection status (self-reported), Q4: Confirmed case, Anti-Spike level | n = 306, Median: 150.0, IQR: (40.05, 250.0), 5%: 7.26, 10%: 12.61 | n = 145, Median: 250.0, IQR: (250.0, 250.0), 5%: 250.0, 10%: 250.0 | n = 202, Median: 5851.5, IQR: (2217.5, 15805.5), 5%: 395.0, 10%: 813.3 | n = 357, Median: 18091.0, IQR: (10524.0, 25000.0), 5%: 5743.2, 10%: 7116.8 |  |  |
| SARS-CoV-2 infection status (self-reported), Q4: Confirmed case, Weeks since vaccination | Median: 7.0, IQR: (5.0, 9.0) | Median: 4.0, IQR: (3.0, 6.0) | Median: 25.0, IQR: (21.0, 29.0) | Median: 5.0, IQR: (3.0, 8.0) |  |  |
| Frailty Index: Healthy, Anti-Spike level | n = 388, Median: 52.1, IQR: (24.83, 130.9), 5%: 5.06, 10%: 10.51 | n = 189, Median: 250.0, IQR: (250.0, 250.0), 5%: 158.22, 10%: 250.0 | n = 241, Median: 1364.0, IQR: (317.0, 5154.0), 5%: 66.4, 10%: 97.7 | n = 503, Median: 14607.0, IQR: (8607.5, 24247.0), 5%: 4473.4, 10%: 5464.6 |  |  |
| Frailty Index: Healthy, Weeks since vaccination | Median: 7.0, IQR: (6.0, 9.0) | Median: 4.0, IQR: (3.0, 5.0) | Median: 24.0, IQR: (19.0, 27.0) | Median: 5.0, IQR: (3.0, 7.0) |  |  |
| Frailty Index: Pre-frail, Anti-Spike level | n = 604, Median: 52.48, IQR: (22.08, 114.2), 5%: 4.74, 10%: 9.87 | n = 320, Median: 250.0, IQR: (250.0, 250.0), 5%: 178.23, 10%: 250.0 | n = 185, Median: 1127.0, IQR: (307.0, 5172.0), 5%: 74.86, 10%: 125.0 | n = 914, Median: 13481.5, IQR: (8037.5, 22984.75), 5%: 3441.1, 10%: 5063.2 |  |  |
| Frailty Index: Pre-frail, Weeks since vaccination | Median: 8.0, IQR: (6.0, 10.0) | Median: 3.0, IQR: (2.0, 5.0) | Median: 26.0, IQR: (23.0, 30.0) | Median: 5.0, IQR: (4.0, 8.0) |  |  |
| Frailty Index: Frail, Anti-Spike level | n = 111, Median: 55.46, IQR: (25.4, 105.4), 5%: 2.69, 10%: 7.75 | n = 90, Median: 250.0, IQR: (250.0, 250.0), 5%: 215.44, 10%: 250.0 | n = 45, Median: 1022.0, IQR: (438.0, 5767.0), 5%: 93.04, 10%: 138.8 | n = 192, Median: 13585.5, IQR: (7378.75, 22779.5), 5%: 3160.1, 10%: 4540.8 |  |  |
| Frailty Index: Frail, Weeks since vaccination | Median: 8.0, IQR: (6.5, 9.0) | Median: 3.0, IQR: (2.0, 5.0) | Median: 29.0, IQR: (26.0, 32.0) | Median: 6.0, IQR: (4.0, 8.0) |  |  |
| Frailty Index: Very frail, Anti-Spike level | n = 35, Median: 48.9, IQR: (20.84, 120.15), 5%: 4.83, 10%: 9.74 | n = 23, Median: 250.0, IQR: (119.25, 250.0), 5%: 7.88, 10%: 36.36 | n = 7, Median: 2444.0, IQR: (884.0, 5069.5), 5%: 469.8, 10%: 528.6 | n = 56, Median: 12848.5, IQR: (6776.75, 22534.0), 5%: 2372.5, 10%: 4015.0 |  |  |
| Frailty Index: Very frail, Weeks since vaccination | Median: 9.0, IQR: (7.0, 10.0) | Median: 4.0, IQR: (2.0, 5.5) | Median: 28.0, IQR: (26.0, 28.5) | Median: 6.0, IQR: (4.75, 8.0) |  |  |
| Frailty (PRISMA-7): Healthy, Anti-Spike level |  |  |  |  | n = 1419, Median: 43.42, IQR: (17.89, 107.55), 5%: 3.24, 10%: 6.55 | n = 274, Median: 250.0, IQR: (250.0, 250.0), 5%: 165.2, 10%: 250.0 |
| Frailty (PRISMA-7): Healthy, Weeks since vaccination |  |  |  |  | Median: 6.0, IQR: (5.0, 8.0) | Median: 4.0, IQR: (2.0, 6.0) |
| Frailty (PRISMA-7): Frail, Anti-Spike level |  |  |  |  | n = 40, Median: 42.0, IQR: (19.33, 70.04), 5%: 2.0, 10%: 4.09 | n = 10, Median: 250.0, IQR: (143.95, 250.0), 5%: 59.11, 10%: 78.79 |
| Frailty (PRISMA-7): Frail, Weeks since vaccination |  |  |  |  | Median: 7.0, IQR: (6.0, 8.0) | Median: 3.0, IQR: (2.25, 4.0) |
| Advised on "Shielded Patient List": No, Anti-Spike level | n = 1292, Median: 54.74, IQR: (23.28, 120.6), 5%: 5.04, 10%: 10.0 | n = 662, Median: 250.0, IQR: (250.0, 250.0), 5%: 179.03, 10%: 250.0 | n = 668, Median: 1316.5, IQR: (333.25, 5187.25), 5%: 73.9, 10%: 112.7 | n = 1746, Median: 13997.5, IQR: (8358.5, 23888.5), 5%: 3970.5, 10%: 5272.0 | n = 1398, Median: 43.82, IQR: (18.26, 107.0), 5%: 3.4, 10%: 6.78 | n = 254, Median: 250.0, IQR: (250.0, 250.0), 5%: 183.01, 10%: 250.0 |
| Advised on "Shielded Patient List": No, Weeks since vaccination | Median: 8.0, IQR: (6.0, 9.0) | Median: 3.0, IQR: (2.0, 5.0) | Median: 25.0, IQR: (20.0, 28.0) | Median: 5.0, IQR: (3.25, 8.0) | Median: 6.0, IQR: (5.0, 8.0) | Median: 4.0, IQR: (2.0, 6.0) |
| Advised on "Shielded Patient List": Yes, Anti-Spike level | n = 82, Median: 43.78, IQR: (13.26, 137.85), 5%: 0.56, 10%: 2.47 | n = 86, Median: 250.0, IQR: (250.0, 250.0), 5%: 24.56, 10%: 56.74 | n = 23, Median: 1801.0, IQR: (385.5, 5767.5), 5%: 62.34, 10%: 113.96 | n = 190, Median: 11244.0, IQR: (6057.5, 19538.0), 5%: 754.5, 10%: 1989.7 | n = 45, Median: 40.75, IQR: (9.39, 64.46), 5%: 0.4, 10%: 0.76 | n = 22, Median: 250.0, IQR: (250.0, 250.0), 5%: 2.35, 10%: 43.13 |
| Advised on "Shielded Patient List": Yes, Weeks since vaccination | Median: 9.0, IQR: (7.0, 10.0) | Median: 3.0, IQR: (2.0, 5.0) | Median: 29.0, IQR: (26.0, 32.0) | Median: 6.5, IQR: (4.0, 8.0) | Median: 8.0, IQR: (7.0, 9.0) | Median: 4.0, IQR: (3.0, 6.75) |
| Prescribed immunosuppressant medication: No, Anti-Spike level | n = 846, Median: 52.0, IQR: (22.6, 117.18), 5%: 5.03, 10%: 10.04 | n = 476, Median: 250.0, IQR: (250.0, 250.0), 5%: 165.75, 10%: 250.0 | n = 357, Median: 1161.0, IQR: (311.0, 4883.0), 5%: 73.48, 10%: 100.88 | n = 1270, Median: 14144.0, IQR: (8210.0, 23532.5), 5%: 3724.75, 10%: 5058.3 |  |  |
| Prescribed immunosuppressant medication: No, Weeks since vaccination | Median: 8.0, IQR: (6.0, 9.0) | Median: 3.0, IQR: (2.0, 5.0) | Median: 25.0, IQR: (21.0, 28.0) | Median: 5.0, IQR: (4.0, 8.0) |  |  |
| Prescribed immunosuppressant medication: Yes, Anti-Spike level | n = 117, Median: 47.94, IQR: (27.12, 112.0), 5%: 3.48, 10%: 10.0 | n = 64, Median: 250.0, IQR: (250.0, 250.0), 5%: 7.32, 10%: 44.74 | n = 37, Median: 1938.0, IQR: (851.0, 7639.0), 5%: 106.0, 10%: 258.4 | n = 192, Median: 12432.0, IQR: (7046.0, 20654.0), 5%: 1371.1, 10%: 3589.1 |  |  |
| Prescribed immunosuppressant medication: Yes, Weeks since vaccination | Median: 9.0, IQR: (7.0, 10.0) | Median: 3.0, IQR: (2.0, 4.25) | Median: 28.0, IQR: (24.0, 32.0) | Median: 5.0, IQR: (4.0, 8.0) |  |  |
| Immunocompromised: No, Anti-Spike level |  |  |  |  | n = 1053, Median: 42.26, IQR: (18.25, 101.0), 5%: 3.39, 10%: 6.59 | n = 198, Median: 250.0, IQR: (250.0, 250.0), 5%: 189.29, 10%: 250.0 |
| Immunocompromised: No, Weeks since vaccination |  |  |  |  | Median: 6.0, IQR: (5.0, 8.0) | Median: 4.0, IQR: (2.0, 6.0) |
| Immunocompromised: Yes, Anti-Spike level |  |  |  |  | n = 39, Median: 38.89, IQR: (6.9, 61.0), 5%: 0.68, 10%: 0.91 | n = 15, Median: 250.0, IQR: (250.0, 250.0), 5%: 27.72, 10%: 90.3 |
| Immunocompromised: Yes, Weeks since vaccination |  |  |  |  | Median: 8.0, IQR: (7.0, 9.0) | Median: 4.0, IQR: (2.0, 4.0) |
| Self-rated health: Poor, Fair, Anti-Spike level | n = 134, Median: 57.2, IQR: (20.61, 118.72), 5%: 1.75, 10%: 3.21 | n = 60, Median: 250.0, IQR: (250.0, 250.0), 5%: 20.31, 10%: 99.87 | n = 42, Median: 1855.5, IQR: (593.5, 16280.0), 5%: 141.4, 10%: 244.5 | n = 168, Median: 13431.0, IQR: (6789.0, 24415.75), 5%: 1237.35, 10%: 4072.7 | n = 137, Median: 39.75, IQR: (20.29, 101.0), 5%: 2.27, 10%: 5.67 | n = 28, Median: 250.0, IQR: (250.0, 250.0), 5%: 77.94, 10%: 140.84 |
| Self-rated health: Poor, Fair, Weeks since vaccination | Median: 9.0, IQR: (7.0, 10.0) | Median: 3.0, IQR: (2.0, 4.25) | Median: 27.0, IQR: (24.25, 29.0) | Median: 6.0, IQR: (4.0, 8.0) | Median: 6.0, IQR: (5.0, 8.0) | Median: 3.5, IQR: (2.75, 7.0) |
| Self-rated health: Good, Very Good, Excellent, Anti-Spike level | n = 1230, Median: 53.1, IQR: (23.0, 121.7), 5%: 5.14, 10%: 10.02 | n = 677, Median: 250.0, IQR: (250.0, 250.0), 5%: 174.24, 10%: 250.0 | n = 614, Median: 1308.5, IQR: (322.75, 5091.75), 5%: 72.67, 10%: 105.3 | n = 1703, Median: 13851.0, IQR: (8243.0, 23522.0), 5%: 3609.5, 10%: 5111.4 | n = 1322, Median: 43.68, IQR: (17.75, 107.18), 5%: 3.37, 10%: 6.52 | n = 255, Median: 250.0, IQR: (250.0, 250.0), 5%: 161.88, 10%: 250.0 |
| Self-rated health: Good, Very Good, Excellent, Weeks since vaccination | Median: 8.0, IQR: (6.0, 9.0) | Median: 3.0, IQR: (2.0, 5.0) | Median: 25.0, IQR: (20.0, 28.0) | Median: 5.0, IQR: (4.0, 8.0) | Median: 6.0, IQR: (5.0, 8.0) | Median: 4.0, IQR: (2.0, 6.0) |
| Number of selected comorbidities: 0, Anti-Spike level | n = 604, Median: 52.52, IQR: (25.51, 125.1), 5%: 4.81, 10%: 9.99 | n = 267, Median: 250.0, IQR: (250.0, 250.0), 5%: 223.01, 10%: 250.0 | n = 308, Median: 1459.0, IQR: (312.5, 5848.25), 5%: 72.04, 10%: 104.1 | n = 789, Median: 13574.0, IQR: (8251.0, 23727.0), 5%: 3872.0, 10%: 5068.8 | n = 744, Median: 42.08, IQR: (17.7, 97.25), 5%: 3.37, 10%: 6.3 | n = 138, Median: 250.0, IQR: (250.0, 250.0), 5%: 189.89, 10%: 250.0 |
| Number of selected comorbidities: 0, Weeks since vaccination | Median: 8.0, IQR: (6.0, 9.0) | Median: 3.0, IQR: (2.0, 5.0) | Median: 25.0, IQR: (20.0, 28.0) | Median: 5.0, IQR: (3.0, 7.0) | Median: 6.0, IQR: (5.0, 7.0) | Median: 3.0, IQR: (2.0, 6.0) |
| Number of selected comorbidities: 1+, Anti-Spike level | n = 452, Median: 51.8, IQR: (20.14, 114.2), 5%: 4.41, 10%: 9.87 | n = 288, Median: 250.0, IQR: (250.0, 250.0), 5%: 97.91, 10%: 248.59 | n = 144, Median: 850.5, IQR: (354.0, 3639.0), 5%: 84.35, 10%: 131.6 | n = 717, Median: 13768.0, IQR: (7833.0, 23953.0), 5%: 3135.4, 10%: 4869.0 | n = 314, Median: 41.8, IQR: (19.28, 108.02), 5%: 3.01, 10%: 8.0 | n = 67, Median: 250.0, IQR: (250.0, 250.0), 5%: 183.75, 10%: 250.0 |
| Number of selected comorbidities: 1+, Weeks since vaccination | Median: 8.0, IQR: (6.0, 10.0) | Median: 3.0, IQR: (2.0, 5.0) | Median: 26.0, IQR: (24.0, 30.0) | Median: 6.0, IQR: (4.0, 8.0) | Median: 7.0, IQR: (5.0, 8.0) | Median: 4.0, IQR: (3.0, 6.5) |
| Comorbidity: Anxiety or Stress Disorder: No, Anti-Spike level | n = 894, Median: 52.29, IQR: (24.12, 125.5), 5%: 4.96, 10%: 10.0 | n = 495, Median: 250.0, IQR: (250.0, 250.0), 5%: 144.44, 10%: 250.0 | n = 371, Median: 1301.0, IQR: (317.5, 5457.5), 5%: 71.55, 10%: 97.7 | n = 1335, Median: 13574.0, IQR: (8055.0, 22890.0), 5%: 3448.1, 10%: 4974.2 | n = 969, Median: 42.55, IQR: (17.74, 100.3), 5%: 3.1, 10%: 6.14 | n = 195, Median: 250.0, IQR: (250.0, 250.0), 5%: 160.26, 10%: 250.0 |
| Comorbidity: Anxiety or Stress Disorder: No, Weeks since vaccination | Median: 8.0, IQR: (6.0, 10.0) | Median: 3.0, IQR: (2.0, 5.0) | Median: 25.0, IQR: (21.0, 29.0) | Median: 5.0, IQR: (4.0, 8.0) | Median: 6.0, IQR: (5.0, 8.0) | Median: 4.0, IQR: (2.0, 6.0) |
| Comorbidity: Anxiety or Stress Disorder: Yes, Anti-Spike level | n = 165, Median: 60.86, IQR: (21.39, 116.8), 5%: 4.28, 10%: 9.5 | n = 81, Median: 250.0, IQR: (250.0, 250.0), 5%: 174.9, 10%: 250.0 | n = 70, Median: 790.0, IQR: (396.5, 3271.0), 5%: 101.35, 10%: 137.5 | n = 213, Median: 15039.0, IQR: (9191.0, 25000.0), 5%: 4215.2, 10%: 5718.2 | n = 114, Median: 39.25, IQR: (19.28, 101.38), 5%: 5.71, 10%: 9.7 | n = 16, Median: 250.0, IQR: (250.0, 250.0), 5%: 227.55, 10%: 250.0 |
| Comorbidity: Anxiety or Stress Disorder: Yes, Weeks since vaccination | Median: 8.0, IQR: (6.0, 9.0) | Median: 3.0, IQR: (2.0, 5.0) | Median: 26.0, IQR: (23.0, 28.0) | Median: 6.0, IQR: (4.0, 7.0) | Median: 6.5, IQR: (6.0, 8.0) | Median: 5.0, IQR: (3.75, 8.0) |
| Comorbidity: Depression: No, Anti-Spike level | n = 917, Median: 54.01, IQR: (24.28, 126.5), 5%: 4.99, 10%: 10.1 | n = 504, Median: 250.0, IQR: (250.0, 250.0), 5%: 151.27, 10%: 250.0 | n = 383, Median: 1301.0, IQR: (321.5, 5602.0), 5%: 67.54, 10%: 98.56 | n = 1353, Median: 13700.0, IQR: (8160.0, 23484.0), 5%: 3685.6, 10%: 5058.2 | n = 1037, Median: 42.55, IQR: (17.82, 100.3), 5%: 3.26, 10%: 6.5 | n = 202, Median: 250.0, IQR: (250.0, 250.0), 5%: 160.32, 10%: 250.0 |
| Comorbidity: Depression: No, Weeks since vaccination | Median: 8.0, IQR: (6.0, 10.0) | Median: 3.0, IQR: (2.0, 5.0) | Median: 25.0, IQR: (21.0, 29.0) | Median: 5.0, IQR: (4.0, 8.0) | Median: 6.0, IQR: (5.0, 8.0) | Median: 4.0, IQR: (2.0, 6.0) |
| Comorbidity: Depression: Yes, Anti-Spike level | n = 134, Median: 48.42, IQR: (20.4, 108.5), 5%: 5.1, 10%: 9.93 | n = 70, Median: 250.0, IQR: (250.0, 250.0), 5%: 98.54, 10%: 170.31 | n = 54, Median: 1157.5, IQR: (377.25, 3339.25), 5%: 121.2, 10%: 242.8 | n = 180, Median: 14426.5, IQR: (7579.75, 24805.5), 5%: 2673.1, 10%: 4952.9 | n = 55, Median: 39.73, IQR: (23.68, 100.94), 5%: 2.92, 10%: 4.4 | n = 9, Median: 250.0, IQR: (250.0, 250.0), 5%: 250.0, 10%: 250.0 |
| Comorbidity: Depression: Yes, Weeks since vaccination | Median: 8.0, IQR: (6.0, 9.0) | Median: 3.0, IQR: (2.0, 5.0) | Median: 26.0, IQR: (22.0, 28.75) | Median: 5.0, IQR: (3.0, 7.0) | Median: 6.0, IQR: (5.0, 7.0) | Median: 4.0, IQR: (3.0, 6.0) |
| Anxiety (HADS) score up to Q2: 8-10, mild, Anti-Spike level | n = 298, Median: 52.36, IQR: (24.94, 120.52), 5%: 6.87, 10%: 10.58 | n = 153, Median: 250.0, IQR: (250.0, 250.0), 5%: 164.98, 10%: 250.0 | n = 143, Median: 808.0, IQR: (318.0, 4259.0), 5%: 94.66, 10%: 146.6 | n = 392, Median: 13739.5, IQR: (7930.5, 24174.25), 5%: 3611.8, 10%: 5222.6 |  |  |
| Anxiety (HADS) score up to Q2: 8-10, mild, Weeks since vaccination | Median: 8.0, IQR: (6.0, 9.0) | Median: 4.0, IQR: (3.0, 5.0) | Median: 25.0, IQR: (20.5, 27.5) | Median: 5.0, IQR: (4.0, 8.0) |  |  |
| Anxiety (HADS) score up to Q2: 11+, moderate, severe, Anti-Spike level | n = 262, Median: 58.33, IQR: (24.18, 125.35), 5%: 4.42, 10%: 10.64 | n = 120, Median: 250.0, IQR: (250.0, 250.0), 5%: 156.53, 10%: 250.0 | n = 137, Median: 1750.0, IQR: (373.0, 6057.0), 5%: 65.62, 10%: 101.8 | n = 276, Median: 14713.0, IQR: (8193.75, 22705.0), 5%: 3705.5, 10%: 5261.0 |  |  |
| Anxiety (HADS) score up to Q2: 11+, moderate, severe, Weeks since vaccination | Median: 8.0, IQR: (6.0, 9.0) | Median: 3.0, IQR: (2.0, 6.0) | Median: 25.0, IQR: (20.0, 28.0) | Median: 6.0, IQR: (4.0, 8.0) |  |  |
| Depression (HADS) score up to Q2: 8-10, mild, Anti-Spike level | n = 227, Median: 56.11, IQR: (25.54, 108.85), 5%: 5.13, 10%: 10.15 | n = 112, Median: 250.0, IQR: (250.0, 250.0), 5%: 119.76, 10%: 243.97 | n = 95, Median: 1301.0, IQR: (314.0, 5997.0), 5%: 72.43, 10%: 121.0 | n = 275, Median: 15186.0, IQR: (8866.5, 23795.0), 5%: 2872.7, 10%: 5317.4 |  |  |
| Depression (HADS) score up to Q2: 8-10, mild, Weeks since vaccination | Median: 8.0, IQR: (6.0, 9.0) | Median: 3.0, IQR: (2.0, 5.0) | Median: 25.0, IQR: (20.0, 29.0) | Median: 5.0, IQR: (3.5, 7.0) |  |  |
| Depression (HADS) score up to Q2: 11+, moderate, severe, Anti-Spike level | n = 151, Median: 58.17, IQR: (25.22, 125.3), 5%: 4.5, 10%: 10.02 | n = 60, Median: 250.0, IQR: (250.0, 250.0), 5%: 242.6, 10%: 250.0 | n = 85, Median: 1057.0, IQR: (311.0, 3963.0), 5%: 67.06, 10%: 90.94 | n = 146, Median: 14243.0, IQR: (7839.0, 24008.75), 5%: 3717.75, 10%: 4951.0 |  |  |
| Depression (HADS) score up to Q2: 11+, moderate, severe, Weeks since vaccination | Median: 8.0, IQR: (6.0, 9.0) | Median: 4.0, IQR: (2.0, 7.0) | Median: 25.0, IQR: (20.0, 29.0) | Median: 5.0, IQR: (3.0, 7.0) |  |  |
| Anxiety (HADS) score up to Q4: 8-10, mild, Anti-Spike level | n = 309, Median: 52.3, IQR: (24.52, 120.6), 5%: 6.97, 10%: 10.6 | n = 160, Median: 250.0, IQR: (250.0, 250.0), 5%: 159.41, 10%: 250.0 | n = 144, Median: 747.0, IQR: (318.0, 3762.75), 5%: 91.02, 10%: 131.6 | n = 431, Median: 13480.0, IQR: (7929.0, 23890.0), 5%: 3646.0, 10%: 5281.0 |  |  |
| Anxiety (HADS) score up to Q4: 8-10, mild, Weeks since vaccination | Median: 8.0, IQR: (6.0, 9.0) | Median: 4.0, IQR: (2.75, 5.0) | Median: 25.0, IQR: (20.0, 27.0) | Median: 5.0, IQR: (4.0, 8.0) |  |  |
| Anxiety (HADS) score up to Q4: 11+, moderate, severe, Anti-Spike level | n = 297, Median: 57.38, IQR: (23.51, 120.6), 5%: 4.37, 10%: 10.04 | n = 138, Median: 250.0, IQR: (250.0, 250.0), 5%: 101.06, 10%: 244.7 | n = 173, Median: 1628.0, IQR: (366.0, 6905.0), 5%: 72.56, 10%: 106.4 | n = 327, Median: 15042.0, IQR: (8254.5, 23350.0), 5%: 3740.7, 10%: 5261.8 |  |  |
| Anxiety (HADS) score up to Q4: 11+, moderate, severe, Weeks since vaccination | Median: 8.0, IQR: (6.0, 9.0) | Median: 3.0, IQR: (2.0, 6.0) | Median: 24.0, IQR: (19.0, 28.0) | Median: 6.0, IQR: (4.0, 8.0) |  |  |
| Depression (HADS) score up to Q4: 8-10, mild, Anti-Spike level | n = 244, Median: 55.09, IQR: (22.38, 110.9), 5%: 3.4, 10%: 8.83 | n = 129, Median: 250.0, IQR: (250.0, 250.0), 5%: 118.82, 10%: 236.86 | n = 106, Median: 1332.5, IQR: (401.5, 5759.5), 5%: 99.3, 10%: 136.0 | n = 331, Median: 14658.0, IQR: (8692.0, 24706.5), 5%: 3202.5, 10%: 5281.0 |  |  |
| Depression (HADS) score up to Q4: 8-10, mild, Weeks since vaccination | Median: 8.0, IQR: (6.0, 9.0) | Median: 3.0, IQR: (2.0, 5.0) | Median: 25.0, IQR: (20.0, 28.0) | Median: 5.0, IQR: (4.0, 7.5) |  |  |
| Depression (HADS) score up to Q4: 11+, moderate, severe, Anti-Spike level | n = 175, Median: 57.51, IQR: (22.77, 122.05), 5%: 4.53, 10%: 10.01 | n = 67, Median: 250.0, IQR: (250.0, 250.0), 5%: 146.4, 10%: 250.0 | n = 105, Median: 1057.0, IQR: (292.0, 4208.0), 5%: 61.84, 10%: 90.94 | n = 174, Median: 14862.5, IQR: (8167.25, 24193.0), 5%: 3838.75, 10%: 5096.7 |  |  |
| Depression (HADS) score up to Q4: 11+, moderate, severe, Weeks since vaccination | Median: 8.0, IQR: (6.0, 9.0) | Median: 4.0, IQR: (2.0, 6.0) | Median: 25.0, IQR: (20.0, 29.0) | Median: 5.0, IQR: (3.0, 7.0) |  |  |
