# Supplementary material for: Antibody levels following vaccination against SARS-CoV-2: associations with post-vaccination infection and risk factors in two UK longitudinal studies
Source: eLife. 2023 Jan 24;12:e80428. doi: 10.7554/eLife.80428 (PMC9940912; doi:10.7554/eLife.80428)
Supplement: Supplementary file 5. [file elife-80428-supp5.docx]

Supplementary file 5. Descriptive statistics relating to post-vaccination infections within TwinsUK, within groups of individuals with varying vaccination status at Q2 and Q4 testing.

| **Vaccination status** | **All who are vaccinated once or more at Q4** | **Single-vaccinated at Q2** | **Double-vaccinated at Q2** |
| --- | --- | --- | --- |
| Q2 anti-Spike antibody level value (BAU/mL): Data not available, n (%) | 267/3539 (7.5%) | 0/1375 (0.0%) | 0/748 (0.0%) |
| Q2 anti-Spike antibody level value (BAU/mL): Median (IQR) | 90.42 (24.65, 250.0) | 53.3 (22.72, 121.2) | 250.0 (250.0, 250.0) |
| Post-vaccination infection between Q2 and Q4 testing: Data not available, n (%) | 570/3539 (16.1%) | 213/1375 (15.5%) | 124/748 (16.6%) |
| Post-vaccination infection between Q2 and Q4 testing: Yes, n (%) | 252/2969 (8.5%) | 105/1162 (9.0%) | 45/624 (7.2%) |
| Post-vaccination infection between Q2 and Q4 testing: No: Q2 Anti-Spike level | Median: 91.56 IQR:(25.46, 250.0), 5%: 0.4, 10%: 0.85 | Median: 57.17 IQR:(23.7, 129.4), 5%: 5.08, 10%: 10.0 | Median: 250.0 IQR:(250.0, 250.0), 5%: 136.47, 10%: 250.0 |
| Post-vaccination infection between Q2 and Q4 testing: Yes: Q2 Anti-Spike level | Median: 48.49 IQR:(8.92, 234.95), 5%: 0.4, 10%: 0.4 | Median: 40.04 IQR:(13.72, 81.82), 5%: 2.54, 10%: 5.11 | Median: 250.0 IQR:(250.0, 250.0), 5%: 149.3, 10%: 240.82 |
| Post-vaccination infection at any time: Data not available, n (%) | 546/3539 (15.4%) | 205/1375 (14.9%) | 114/748 (15.2%) |
| Post-vaccination infection at any time: Yes, n (%) | 276/2993 (9.2%) | 113/1170 (9.7%) | 55/634 (8.7%) |
| Post-vaccination infection, exact date of infection unknown: Yes, n | 32 | 9 | 6 |
| Post-vaccination infection while single-vaccinated: Yes, n | 34 | 12 | 8 |
| Post-vaccination infection while double-vaccinated: Yes, n | 171 | 83 | 29 |
| Post-vaccination infection while triple-vaccinated: Yes, n | 39 | 9 | 12 |
| Post-vaccination infection while single-vaccinated: Weeks since first vaccination: Median (IQR) | 5.5 (1.25, 9.0) | 4.0 (1.75, 9.5) | 1.0 (0.0, 4.0) |
| Post-vaccination infection while double-vaccinated: Weeks since second vaccination: Median (IQR) | 19.0 (11.5, 23.0) | 19.0 (11.0, 23.0) | 23.0 (20.0, 26.0) |
| Post-vaccination infection while triple-vaccinated: Weeks since third vaccination: Median (IQR) | 5.0 (1.0, 7.5) | 1.0 (1.0, 2.0) | 9.5 (7.75, 10.5) |
| Post-vaccination infection while single-vaccinated: Days since first vaccination: Median (IQR) | 41.5 (12.25, 64.75) | 29.5 (14.75, 68.0) | 7.5 (4.25, 28.5) |
| Post-vaccination infection while double-vaccinated: Days since second vaccination: Median (IQR) | 136.0 (83.5, 167.0) | 136.0 (78.5, 164.5) | 167.0 (143.0, 187.0) |
| Post-vaccination infection while triple-vaccinated: Days since third vaccination: Median (IQR) | 37.0 (9.0, 57.0) | 10.0 (9.0, 16.0) | 69.0 (57.75, 78.0) |
| Post-vaccination infection while single-vaccinated: Date & likely variant: 1. Before May 2021: Alpha, n | 22 | 8 | 8 |
| Post-vaccination infection while single-vaccinated: Date & likely variant: 2. May-Dec 2021: Delta, n | 9 | < 5 | < 5 |
| Post-vaccination infection while single-vaccinated: Date & likely variant: 3. After Dec 2021: Omicron, n | < 5 | < 5 | < 5 |
| Post-vaccination infection while double-vaccinated: Date & likely variant: 1. Before May 2021: Alpha, n | < 5 | < 5 | < 5 |
| Post-vaccination infection while double-vaccinated: Date & likely variant: 2. May-Dec 2021: Delta, n | 158 | 80 | 26 |
| Post-vaccination infection while double-vaccinated: Date & likely variant: 3. After Dec 2021: Omicron, n | 11 | < 5 | < 5 |
| Post-vaccination infection while triple-vaccinated: Date & likely variant: 1. Before May 2021: Alpha, n | < 5 | < 5 | < 5 |
| Post-vaccination infection while triple-vaccinated: Date & likely variant: 2. May-Dec 2021: Delta, n | 17 | 5 | < 5 |
| Post-vaccination infection while triple-vaccinated: Date & likely variant: 3. After Dec 2021: Omicron, n | 22 | < 5 | 9 |
| Post-vaccination infection while single-vaccinated: UK 7-day rolling case rate at time of infection: Median (IQR) | 104.45 (57.5, 387.5) | 57.05 (37.97, 65.22) | 236.05 (130.65, 525.28) |
| Post-vaccination infection while double-vaccinated: UK 7-day rolling case rate at time of infection: Median (IQR) | 373.1 (350.1, 442.85) | 392.6 (352.5, 443.45) | 362.5 (309.9, 406.4) |
| Post-vaccination infection while triple-vaccinated: UK 7-day rolling case rate at time of infection: Median (IQR) | 507.5 (444.35, 1214.65) | 448.7 (445.1, 530.5) | 1017.05 (472.18, 1697.02) |
