# Supplementary material for: Antibody levels following vaccination against SARS-CoV-2: associations with post-vaccination infection and risk factors in two UK longitudinal studies
Source: eLife. 2023 Jan 24;12:e80428. doi: 10.7554/eLife.80428 (PMC9940912; doi:10.7554/eLife.80428)
Supplement: Supplementary file 6. — Results present odds ratios, unadjusted 95% confidence intervals, and p-values adjusted for multiple testing. Results based on fewer than three individuals having post-vaccination infection are suppressed. Variables with adjusted p-values <0.05 are highlighted in bold. [file elife-80428-supp6.docx]

Supplementary file 6. Logistic regression model results, testing for association between post-vaccination infection and socio-demographic, SARS-CoV-2 vaccination, and SARS-CoV-2 infection variables for TwinsUK individuals who participated in antibody testing at Q2 and one or both of Q4 antibody testing and Q4 questionnaire, who reported one or more vaccination reported by Q4. Results present odds ratios, unadjusted 95% confidence intervals, and p-values adjusted for multiple testing. Results based on fewer than 3 individuals having post-vaccination infection are suppressed. Variables with adjusted p-values < 0.05 are highlighted in bold.

| **Control variables** | **Domain** | **Test variable** | **Outcome: post-vaccination infection at any time** |
| --- | --- | --- | --- |
| Adjusted for: Age | COVID-19 infection | Anti-Nucleocapsid antibody status: Positive | 0.55 (0.34, 0.89), p = 0.17 |
| Adjusted for: Age | COVID-19 infection | SARS-CoV-2 infection status (self-reported): Confirmed case | 0.92 (0.64, 1.32), p = 0.86 |
| Adjusted for: Age | COVID-19 infection | SARS-CoV-2 infection status (self-reported): Suspected case | 0.73 (0.47, 1.13), p = 0.75 |
| Adjusted for: Age | COVID-19 infection | SARS-CoV-2 infection status (self-reported): Suspected or confirmed case | 0.84 (0.62, 1.13), p = 0.75 |
| Adjusted for: Age | COVID-19 infection | SARS-CoV-2 infection status (self-reported): Unsure | 1.26 (0.56, 2.84), p = 0.86 |
| Adjusted for: Age | COVID-19 infection | SARS-CoV-2 infection status (serology-based): Evidence of natural infection | **0.46 (0.32, 0.67), p = 0.0009** |
| Adjusted for: Age | COVID-19 vaccination | First vaccination received: Other | 0.83 (0.35, 1.97), p = 0.87 |
| Adjusted for: Age | COVID-19 vaccination | First vaccination received: AZD1222 (Oxford/AZ) | 1.36 (1.03, 1.78), p = 0.27 |
| Adjusted for: Age | Socio-demographics | Deprivation (IMD): Most deprived 40% (decile 1-4) | 0.91 (0.65, 1.27), p = 0.86 |
| Adjusted for: Age | Socio-demographics | Employment status: In education | 0.82 (0.35, 1.9), p = 0.86 |
| Adjusted for: Age | Socio-demographics | Employment status: Looking after home or family (unpaid care) | 0.8 (0.4, 1.59), p = 0.85 |
| Adjusted for: Age | Socio-demographics | Employment status: Maternity leave | Suppressed |
| Adjusted for: Age | Socio-demographics | Employment status: Other | Suppressed |
| Adjusted for: Age | Socio-demographics | Employment status: Permanently (or long-term) sick or disabled | Suppressed |
| Adjusted for: Age | Socio-demographics | Employment status: Retired | **0.5 (0.34, 0.75), p = 0.01** |
| Adjusted for: Age | Socio-demographics | Employment status: Self-employed | 0.88 (0.55, 1.4), p = 0.86 |
| Adjusted for: Age | Socio-demographics | Employment status: Semi-retired/part-time employment | 1.89 (0.53, 6.75), p = 0.81 |
| Adjusted for: Age | Socio-demographics | Employment status: Unemployed | Suppressed |
| Adjusted for: Age | Socio-demographics | Employment status: Unpaid/voluntary work | 0.36 (0.11, 1.17), p = 0.69 |
| Adjusted for: Age | Socio-demographics | Ethnicity: Other than white | 0.99 (0.47, 2.1), p = 0.99 |
| Adjusted for: Age | Socio-demographics | IMD: -1 decile (increasing deprivation) | 0.98 (0.93, 1.03), p = 0.81 |
| Adjusted for: Age | Socio-demographics | Highest educational attainment: NVQ level 3 or lower | 1.13 (0.85, 1.49), p = 0.81 |
| Adjusted for: Age | Socio-demographics | RUC: Urban | 1.01 (0.76, 1.35), p = 0.97 |
| Adjusted for: Age, SARS-CoV-2 infection status (serology-based) | COVID-19 infection | Anti-Nucleocapsid antibody status: Positive | 1.27 (0.63, 2.58), p = 0.83 |
| Adjusted for: Age, SARS-CoV-2 infection status (serology-based) | COVID-19 infection | SARS-CoV-2 infection status (self-reported): Confirmed case | 1.66 (1.07, 2.57), p = 0.32 |
| Adjusted for: Age, SARS-CoV-2 infection status (serology-based) | COVID-19 infection | SARS-CoV-2 infection status (self-reported): Suspected case | 0.78 (0.5, 1.21), p = 0.83 |
| Adjusted for: Age, SARS-CoV-2 infection status (serology-based) | COVID-19 infection | SARS-CoV-2 infection status (self-reported): Suspected or confirmed case | 1.11 (0.81, 1.53), p = 0.84 |
| Adjusted for: Age, SARS-CoV-2 infection status (serology-based) | COVID-19 infection | SARS-CoV-2 infection status (self-reported): Unsure | 1.28 (0.56, 2.9), p = 0.84 |
| Adjusted for: Age, SARS-CoV-2 infection status (serology-based) | COVID-19 vaccination | First vaccination received: Other | 0.85 (0.35, 2.08), p = 0.92 |
| Adjusted for: Age, SARS-CoV-2 infection status (serology-based) | COVID-19 vaccination | First vaccination received: AZD1222 (Oxford/AZ) | 1.29 (0.98, 1.69), p = 0.75 |
| Adjusted for: Age, SARS-CoV-2 infection status (serology-based) | Socio-demographics | Deprivation (IMD): Most deprived 40% (decile 1-4) | 0.92 (0.65, 1.29), p = 0.84 |
| Adjusted for: Age, SARS-CoV-2 infection status (serology-based) | Socio-demographics | Employment status: In education | 0.76 (0.32, 1.85), p = 0.84 |
| Adjusted for: Age, SARS-CoV-2 infection status (serology-based) | Socio-demographics | Employment status: Looking after home or family (unpaid care) | 0.82 (0.41, 1.62), p = 0.84 |
| Adjusted for: Age, SARS-CoV-2 infection status (serology-based) | Socio-demographics | Employment status: Maternity leave | Suppressed |
| Adjusted for: Age, SARS-CoV-2 infection status (serology-based) | Socio-demographics | Employment status: Other | Suppressed |
| Adjusted for: Age, SARS-CoV-2 infection status (serology-based) | Socio-demographics | Employment status: Permanently (or long-term) sick or disabled | Suppressed |
| Adjusted for: Age, SARS-CoV-2 infection status (serology-based) | Socio-demographics | Employment status: Retired | **0.49 (0.33, 0.74), p = 0.01** |
| Adjusted for: Age, SARS-CoV-2 infection status (serology-based) | Socio-demographics | Employment status: Self-employed | 0.83 (0.52, 1.32), p = 0.83 |
| Adjusted for: Age, SARS-CoV-2 infection status (serology-based) | Socio-demographics | Employment status: Semi-retired/part-time employment | 1.84 (0.52, 6.54), p = 0.83 |
| Adjusted for: Age, SARS-CoV-2 infection status (serology-based) | Socio-demographics | Employment status: Unemployed | Suppressed |
| Adjusted for: Age, SARS-CoV-2 infection status (serology-based) | Socio-demographics | Employment status: Unpaid/voluntary work | 0.36 (0.11, 1.18), p = 0.75 |
| Adjusted for: Age, SARS-CoV-2 infection status (serology-based) | Socio-demographics | Ethnicity: Other than white | 1.09 (0.51, 2.35), p = 0.93 |
| Adjusted for: Age, SARS-CoV-2 infection status (serology-based) | Socio-demographics | IMD: -1 decile (increasing deprivation) | 0.98 (0.93, 1.03), p = 0.83 |
| Adjusted for: Age, SARS-CoV-2 infection status (serology-based) | Socio-demographics | Highest educational attainment: NVQ level 3 or lower | 1.15 (0.87, 1.52), p = 0.83 |
| Adjusted for: Age, SARS-CoV-2 infection status (serology-based) | Socio-demographics | RUC: Urban | 1.03 (0.77, 1.38), p = 0.93 |
| Adjusted for: Age, Sex, SARS-CoV-2 infection status (serology-based) | COVID-19 infection | Anti-Nucleocapsid antibody status: Positive | 1.28 (0.63, 2.59), p = 0.86 |
| Adjusted for: Age, Sex, SARS-CoV-2 infection status (serology-based) | COVID-19 infection | SARS-CoV-2 infection status (self-reported): Confirmed case | 1.67 (1.08, 2.6), p = 0.26 |
| Adjusted for: Age, Sex, SARS-CoV-2 infection status (serology-based) | COVID-19 infection | SARS-CoV-2 infection status (self-reported): Suspected case | 0.79 (0.51, 1.23), p = 0.84 |
| Adjusted for: Age, Sex, SARS-CoV-2 infection status (serology-based) | COVID-19 infection | SARS-CoV-2 infection status (self-reported): Suspected or confirmed case | 1.13 (0.82, 1.55), p = 0.86 |
| Adjusted for: Age, Sex, SARS-CoV-2 infection status (serology-based) | COVID-19 infection | SARS-CoV-2 infection status (self-reported): Unsure | 1.28 (0.56, 2.93), p = 0.86 |
| Adjusted for: Age, Sex, SARS-CoV-2 infection status (serology-based) | COVID-19 vaccination | First vaccination received: Other | 0.8 (0.33, 1.95), p = 0.87 |
| Adjusted for: Age, Sex, SARS-CoV-2 infection status (serology-based) | COVID-19 vaccination | First vaccination received: AZD1222 (Oxford/AZ) | 1.28 (0.98, 1.68), p = 0.63 |
| Adjusted for: Age, Sex, SARS-CoV-2 infection status (serology-based) | Socio-demographics | Deprivation (IMD): Most deprived 40% (decile 1-4) | 0.92 (0.65, 1.29), p = 0.87 |
| Adjusted for: Age, Sex, SARS-CoV-2 infection status (serology-based) | Socio-demographics | Employment status: In education | 0.76 (0.31, 1.85), p = 0.86 |
| Adjusted for: Age, Sex, SARS-CoV-2 infection status (serology-based) | Socio-demographics | Employment status: Looking after home or family (unpaid care) | 0.86 (0.43, 1.71), p = 0.87 |
| Adjusted for: Age, Sex, SARS-CoV-2 infection status (serology-based) | Socio-demographics | Employment status: Maternity leave | Suppressed |
| Adjusted for: Age, Sex, SARS-CoV-2 infection status (serology-based) | Socio-demographics | Employment status: Other | Suppressed |
| Adjusted for: Age, Sex, SARS-CoV-2 infection status (serology-based) | Socio-demographics | Employment status: Permanently (or long-term) sick or disabled | Suppressed |
| Adjusted for: Age, Sex, SARS-CoV-2 infection status (serology-based) | Socio-demographics | Employment status: Retired | **0.5 (0.33, 0.74), p = 0.01** |
| Adjusted for: Age, Sex, SARS-CoV-2 infection status (serology-based) | Socio-demographics | Employment status: Self-employed | 0.82 (0.51, 1.31), p = 0.85 |
| Adjusted for: Age, Sex, SARS-CoV-2 infection status (serology-based) | Socio-demographics | Employment status: Semi-retired/part-time employment | 1.73 (0.51, 5.93), p = 0.85 |
| Adjusted for: Age, Sex, SARS-CoV-2 infection status (serology-based) | Socio-demographics | Employment status: Unemployed | Suppressed |
| Adjusted for: Age, Sex, SARS-CoV-2 infection status (serology-based) | Socio-demographics | Employment status: Unpaid/voluntary work | 0.36 (0.11, 1.19), p = 0.68 |
| Adjusted for: Age, Sex, SARS-CoV-2 infection status (serology-based) | Socio-demographics | Ethnicity: Other than white | 1.11 (0.51, 2.39), p = 0.93 |
| Adjusted for: Age, Sex, SARS-CoV-2 infection status (serology-based) | Socio-demographics | IMD: -1 decile (increasing deprivation) | 0.98 (0.93, 1.03), p = 0.86 |
| Adjusted for: Age, Sex, SARS-CoV-2 infection status (serology-based) | Socio-demographics | Highest educational attainment: NVQ level 3 or lower | 1.19 (0.89, 1.57), p = 0.84 |
| Adjusted for: Age, Sex, SARS-CoV-2 infection status (serology-based) | Socio-demographics | RUC: Urban | 1.02 (0.76, 1.37), p = 0.99 |
| Adjusted for: Age, Sex, SARS-CoV-2 infection status (serology-based) | Socio-demographics | Sex: Male | 1.49 (1.06, 2.12), p = 0.26 |
| No control variables | COVID-19 infection | Anti-Nucleocapsid antibody status: Positive | 0.71 (0.45, 1.12), p = 0.42 |
| No control variables | COVID-19 infection | SARS-CoV-2 infection status (self-reported): Confirmed case | 1.05 (0.74, 1.48), p = 0.89 |
| No control variables | COVID-19 infection | SARS-CoV-2 infection status (self-reported): Suspected case | 0.81 (0.53, 1.25), p = 0.67 |
| No control variables | COVID-19 infection | SARS-CoV-2 infection status (self-reported): Suspected or confirmed case | 0.95 (0.71, 1.26), p = 0.86 |
| No control variables | COVID-19 infection | SARS-CoV-2 infection status (self-reported): Unsure | 1.46 (0.65, 3.27), p = 0.68 |
| No control variables | COVID-19 infection | SARS-CoV-2 infection status (serology-based): Evidence of natural infection | **0.53 (0.37, 0.77), p = 0.006** |
| No control variables | COVID-19 vaccination | First vaccination received: Other | 1.4 (0.58, 3.35), p = 0.75 |
| No control variables | COVID-19 vaccination | First vaccination received: AZD1222 (Oxford/AZ) | 1.18 (0.91, 1.53), p = 0.52 |
| No control variables | Socio-demographics | Age: 50-59 | 0.94 (0.68, 1.31), p = 0.86 |
| No control variables | Socio-demographics | Age: 60-69 | **0.49 (0.35, 0.69), p = 0.0007** |
| No control variables | Socio-demographics | Age: 70-79 | **0.31 (0.21, 0.45), p < 0.0001** |
| No control variables | Socio-demographics | Age: 80+ | **0.18 (0.07, 0.44), p = 0.002** |
| No control variables | Socio-demographics | Deprivation (IMD): Most deprived 40% (decile 1-4) | 1.0 (0.72, 1.39), p = 0.99 |
| No control variables | Socio-demographics | Employment status: In education | 1.16 (0.51, 2.63), p = 0.86 |
| No control variables | Socio-demographics | Employment status: Looking after home or family (unpaid care) | 0.69 (0.35, 1.35), p = 0.59 |
| No control variables | Socio-demographics | Employment status: Maternity leave | Suppressed |
| No control variables | Socio-demographics | Employment status: Other | Suppressed |
| No control variables | Socio-demographics | Employment status: Permanently (or long-term) sick or disabled | Suppressed |
| No control variables | Socio-demographics | Employment status: Retired | **0.36 (0.27, 0.49), p < 0.0001** |
| No control variables | Socio-demographics | Employment status: Self-employed | 0.8 (0.51, 1.26), p = 0.66 |
| No control variables | Socio-demographics | Employment status: Semi-retired/part-time employment | 1.41 (0.4, 4.98), p = 0.83 |
| No control variables | Socio-demographics | Employment status: Unemployed | Suppressed |
| No control variables | Socio-demographics | Employment status: Unpaid/voluntary work | 0.28 (0.09, 0.9), p = 0.12 |
| No control variables | Socio-demographics | Ethnicity: Other than white | 1.35 (0.64, 2.85), p = 0.75 |
| No control variables | Socio-demographics | IMD: -1 decile (increasing deprivation) | 0.99 (0.94, 1.05), p = 0.9 |
| No control variables | Socio-demographics | Highest educational attainment: NVQ level 3 or lower | 0.85 (0.65, 1.11), p = 0.55 |
| No control variables | Socio-demographics | RUC: Urban | 1.11 (0.83, 1.47), p = 0.76 |
