# Supplementary material for: Antibody levels following vaccination against SARS-CoV-2: associations with post-vaccination infection and risk factors in two UK longitudinal studies
Source: eLife. 2023 Jan 24;12:e80428. doi: 10.7554/eLife.80428 (PMC9940912; doi:10.7554/eLife.80428)
Supplement: Supplementary file 7. — Results present odds ratios, unadjusted 95% confidence intervals, and p-values adjusted for multiple testing. Results based on fewer than three individuals being in the low antibody level group are suppressed. Sets of adjustment variables included in addition to the exposure variable in each model were age, sex, most recent vaccine received and weeks since most recent vaccination, aside from cases where the effect of adjustment variables were themselves tested. In these cases, all other adjustment variables within the given set were included in addition to the adjustment variable being tested. Variables with adjusted p-values <0.05 are highlighted in bold. [file elife-80428-supp7.docx]

Supplementary file 7. Logistic regression model results, testing for association with low anti-Spike antibody levels after first, second and third SARS-CoV-2 vaccination within TwinsUK and ALSPAC at Q2 or Q4 testing. Results present odds ratios, unadjusted 95% confidence intervals, and p-values adjusted for multiple testing. Results based on fewer than 3 individuals being in the low antibody level group are suppressed. Sets of adjustment variables included in addition to the exposure variable in each model were age, sex, most recent vaccine received and weeks since most recent vaccination, aside from cases where the effect of adjustment variables were themselves tested. In these cases, all other adjustment variables within the given set were included in addition to the adjustment variable being tested. Variables with adjusted p-values < 0.05 are highlighted in bold.

|  | **Cohort** | **TwinsUK** | | | | **ALSPAC** | |
| --- | --- | --- | --- | --- | --- | --- | --- |
|  | **Testing period** | **Q2** | | **Q4** | | **Q2** | |
|  | **Number of vaccinations received** | **1** | **2** | **2** | **3** | **1** | **2** |
|  | **Outcome threshold** | **Lowest 10%** | **Lowest 8%** | **Lowest 10%** | **Lowest 10%** | **Lowest 10%** | **Lowest 8%** |
| **Variable group** | **Variable** |  |  |  |  |  |  |
| COVID-19 at-risk | Advised on "Shielded Patient List": Yes | **4.03 (2.2, 7.42), p = 0.0001** | **3.0 (1.55, 5.8), p = 0.009** | 0.96 (0.21, 4.36), p = 0.96 | **2.42 (1.62, 3.64), p = 0.0002** | **4.13 (1.79, 9.51), p = 0.02** | 2.23 (0.58, 8.65), p = 0.78 |
| COVID-19 at-risk | Frailty (PRISMA-7 assessment): Above threshold |  |  |  |  | 1.78 (0.71, 4.43), p = 0.55 | 1.95 (0.34, 11.37), p = 0.78 |
| COVID-19 at-risk | Frailty Index: 2. Pre-frail | 1.22 (0.83, 1.78), p = 0.62 | 0.83 (0.44, 1.59), p = 0.7 | 0.69 (0.37, 1.32), p = 0.54 | 0.92 (0.64, 1.32), p = 0.87 |  |  |
| COVID-19 at-risk | Frailty Index: 3. Frail | 1.5 (0.77, 2.93), p = 0.55 | 0.62 (0.24, 1.63), p = 0.55 | 0.57 (0.18, 1.79), p = 0.59 | 1.13 (0.66, 1.93), p = 0.87 |  |  |
| COVID-19 at-risk | Frailty Index: 4. Very frail | 1.39 (0.39, 4.94), p = 0.75 | **6.64 (2.32, 18.99), p = 0.004** | Suppressed | 1.81 (0.83, 3.94), p = 0.4 |  |  |
| COVID-19 at-risk | Prescribed immunosuppressant medication | 1.11 (0.55, 2.24), p = 0.87 | **3.65 (1.77, 7.51), p = 0.004** | 0.78 (0.2, 2.99), p = 0.85 | 1.46 (0.91, 2.37), p = 0.38 |  |  |
| COVID-19 at-risk | Self-reported immunosuppressed |  |  |  |  | **6.2 (2.65, 14.5), p = 0.001** | 1.82 (0.36, 9.17), p = 0.78 |
| COVID-19 infection | Anti-Nucleocapsid antibody status: Positive | Suppressed | Suppressed | Suppressed | **0.1 (0.04, 0.29), p = 0.0002** | Suppressed | Suppressed |
| COVID-19 infection | SARS-CoV-2 infection status (self-reported): Confirmed case | **0.28 (0.13, 0.59), p = 0.007** | Suppressed | Suppressed | **0.25 (0.13, 0.45), p = 0.0001** | Suppressed | Suppressed |
| COVID-19 infection | SARS-CoV-2 infection status (self-reported): Suspected case | 1.16 (0.71, 1.9), p = 0.72 | 0.77 (0.26, 2.29), p = 0.74 | 1.18 (0.57, 2.41), p = 0.82 | 0.82 (0.5, 1.35), p = 0.79 | 0.81 (0.5, 1.32), p = 0.78 | 0.61 (0.16, 2.43), p = 0.78 |
| COVID-19 infection | SARS-CoV-2 infection status (self-reported): Suspected or confirmed case | 0.65 (0.42, 0.99), p = 0.23 | 0.35 (0.14, 0.86), p = 0.09 | **0.3 (0.16, 0.57), p = 0.001** | **0.44 (0.3, 0.67), p = 0.0008** | 0.69 (0.42, 1.12), p = 0.54 | 0.56 (0.14, 2.18), p = 0.78 |
| COVID-19 infection | SARS-CoV-2 infection status (self-reported): Unsure | 0.6 (0.19, 1.93), p = 0.63 | Suppressed | Suppressed | 0.68 (0.28, 1.65), p = 0.77 |  |  |
| COVID-19 infection | SARS-CoV-2 infection status (serology-based): Evidence of natural infection | **0.49 (0.29, 0.82), p = 0.04** | 0.58 (0.26, 1.32), p = 0.36 | **0.09 (0.03, 0.26), p < 0.0001** | **0.45 (0.28, 0.71), p = 0.004** | Suppressed | Suppressed |
| COVID-19 vaccination | First vaccination received: Other | Suppressed |  |  |  |  |  |
| COVID-19 vaccination | First vaccination received: AZD1222 | **3.05 (1.46, 6.39), p = 0.02** |  |  |  | 3.23 (1.36, 7.68), p = 0.09 |  |
| COVID-19 vaccination | Second vaccination received: Other |  | Suppressed | Suppressed | Suppressed |  |  |
| COVID-19 vaccination | Second vaccination received: AZD1222 |  | **4.65 (2.44, 8.86), p < 0.0001** | **45.69 (5.61, 372.28), p = 0.001** | 1.13 (0.82, 1.56), p = 0.8 |  | **20.34 (6.39, 64.68), p < 0.0001** |
| COVID-19 vaccination | Third vaccination received: mRNA-1273 |  |  |  | 0.34 (0.13, 0.86), p = 0.11 |  |  |
| COVID-19 vaccination | Third vaccination received: Other |  |  |  | Suppressed |  |  |
| COVID-19 vaccination | Weeks since first vaccination: +1 week | **0.83 (0.75, 0.92), p = 0.007** |  |  |  | 0.83 (0.73, 0.95), p = 0.09 |  |
| COVID-19 vaccination | Weeks since second vaccination: +1 week |  | **1.14 (1.04, 1.24), p = 0.02** | 1.02 (0.96, 1.09), p = 0.7 |  |  | 1.13 (0.97, 1.32), p = 0.78 |
| COVID-19 vaccination | Weeks since third vaccination: +1 week |  |  |  | **1.24 (1.18, 1.29), p < 0.0001** |  |  |
| Comorbidities | # Selected comorbidities: 1 | 1.23 (0.8, 1.88), p = 0.63 | 1.19 (0.64, 2.21), p = 0.7 | 0.72 (0.35, 1.49), p = 0.62 | 0.99 (0.68, 1.43), p = 0.95 | 0.92 (0.56, 1.51), p = 0.87 | 0.45 (0.08, 2.44), p = 0.78 |
| Comorbidities | # Selected comorbidities: 2 | 0.89 (0.37, 2.14), p = 0.89 | 0.88 (0.34, 2.26), p = 0.86 | Suppressed | 0.92 (0.51, 1.66), p = 0.92 | 1.43 (0.48, 4.28), p = 0.79 | 3.18 (0.5, 20.04), p = 0.78 |
| Comorbidities | # Selected comorbidities: 3 | 4.27 (1.13, 16.07), p = 0.17 | **5.21 (1.5, 18.02), p = 0.04** | Suppressed | 2.78 (1.09, 7.12), p = 0.14 | Suppressed | Suppressed |
| Comorbidities | # Selected comorbidities: 4+ | Suppressed |  |  | Suppressed | Suppressed |  |
| Comorbidities | Comorbidity domain: Arthritis (any) | 1.56 (1.0, 2.45), p = 0.24 | 1.16 (0.62, 2.16), p = 0.74 | 0.72 (0.28, 1.85), p = 0.7 | 1.36 (0.96, 1.94), p = 0.32 |  |  |
| Comorbidities | Comorbidity domain: Cardiac Disease | 1.62 (0.65, 4.07), p = 0.62 | 1.51 (0.61, 3.72), p = 0.6 | Suppressed | 0.74 (0.37, 1.47), p = 0.77 |  |  |
| Comorbidities | Comorbidity domain: Cardiac Risk Factors | 0.82 (0.53, 1.27), p = 0.63 | 1.5 (0.85, 2.66), p = 0.34 | 0.55 (0.23, 1.29), p = 0.44 | 1.06 (0.76, 1.49), p = 0.91 |  |  |
| Comorbidities | Comorbidity domain: Neurological Disease | 1.24 (0.77, 1.99), p = 0.63 | 1.35 (0.67, 2.72), p = 0.61 | 0.56 (0.24, 1.28), p = 0.44 | 0.96 (0.62, 1.48), p = 0.92 | Suppressed | Suppressed |
| Comorbidities | Comorbidity domain: Subjective Memory Impairment | 0.64 (0.3, 1.34), p = 0.55 | 1.04 (0.34, 3.19), p = 0.95 | 1.02 (0.37, 2.84), p = 0.96 | 1.29 (0.72, 2.32), p = 0.77 |  |  |
| Comorbidities | Comorbidity: Anxiety or Depression | 1.05 (0.65, 1.7), p = 0.93 | 1.46 (0.74, 2.89), p = 0.48 | 0.54 (0.23, 1.24), p = 0.43 | 1.06 (0.69, 1.62), p = 0.92 | 0.81 (0.42, 1.58), p = 0.79 | Suppressed |
| Comorbidities | Comorbidity: Anxiety or Stress Disorder | 1.13 (0.65, 1.97), p = 0.8 | 0.7 (0.27, 1.86), p = 0.65 | 0.61 (0.23, 1.61), p = 0.57 | 0.72 (0.41, 1.25), p = 0.62 | 0.59 (0.26, 1.33), p = 0.55 | Suppressed |
| Comorbidities | Comorbidity: Asthma | 0.98 (0.57, 1.69), p = 0.95 | 2.09 (1.05, 4.14), p = 0.11 | 0.7 (0.31, 1.6), p = 0.63 | 1.26 (0.83, 1.93), p = 0.64 | 1.29 (0.63, 2.64), p = 0.79 | Suppressed |
| Comorbidities | Comorbidity: Atrial Fibrillation | Suppressed | 0.96 (0.27, 3.41), p = 0.95 | Suppressed | 0.43 (0.16, 1.17), p = 0.34 |  |  |
| Comorbidities | Comorbidity: Cancer (any) | 1.34 (0.69, 2.63), p = 0.63 | 2.13 (1.05, 4.31), p = 0.11 | 0.74 (0.2, 2.71), p = 0.82 | 1.37 (0.85, 2.2), p = 0.52 | Suppressed | Suppressed |
| Comorbidities | Comorbidity: Lung disease | 1.42 (0.43, 4.68), p = 0.72 | 2.87 (0.88, 9.34), p = 0.2 | Suppressed | 1.16 (0.51, 2.66), p = 0.91 | Suppressed | Suppressed |
| Comorbidities | Comorbidity: Depression | 0.98 (0.53, 1.8), p = 0.95 | 1.86 (0.82, 4.2), p = 0.3 | 0.41 (0.12, 1.4), p = 0.44 | 1.2 (0.71, 2.03), p = 0.84 | 1.23 (0.53, 2.86), p = 0.85 | Suppressed |
| Comorbidities | Comorbidity: Diabetes (any) | 1.49 (0.42, 5.31), p = 0.72 | 1.26 (0.37, 4.33), p = 0.79 | Suppressed | 0.52 (0.15, 1.83), p = 0.69 | 2.1 (0.68, 6.49), p = 0.55 | Suppressed |
| Comorbidities | Comorbidity: Heart disease (CHD or Heart failure) | Suppressed | Suppressed |  | 1.16 (0.35, 3.79), p = 0.92 | Suppressed | 2.75 (0.43, 17.7), p = 0.78 |
| Comorbidities | Comorbidity: High Cholesterol | 0.81 (0.48, 1.35), p = 0.65 | 1.56 (0.85, 2.87), p = 0.33 | Suppressed | 0.92 (0.63, 1.33), p = 0.87 |  |  |
| Comorbidities | Comorbidity: Hypertension | 1.4 (0.86, 2.29), p = 0.49 | 0.81 (0.42, 1.58), p = 0.68 | 0.87 (0.28, 2.68), p = 0.89 | 1.04 (0.71, 1.53), p = 0.92 | 1.25 (0.72, 2.18), p = 0.78 | 1.32 (0.33, 5.34), p = 0.88 |
| Comorbidities | Comorbidity: Osteoporosis | 1.06 (0.53, 2.1), p = 0.94 | 1.66 (0.79, 3.5), p = 0.35 | Suppressed | 1.18 (0.75, 1.85), p = 0.8 |  |  |
| Comorbidities | Comorbidity: Rheumatoid Arthritis | 1.61 (0.63, 4.12), p = 0.62 | 2.39 (0.57, 10.02), p = 0.42 | Suppressed | **3.03 (1.35, 6.82), p = 0.04** |  |  |
| Comorbidities | Comorbidity: Stroke | 3.2 (0.95, 10.72), p = 0.26 | Suppressed | Suppressed | 0.88 (0.26, 3.06), p = 0.92 |  |  |
| General health | BMI: +1 kg/m^2 | 1.03 (0.99, 1.07), p = 0.4 | 1.03 (0.96, 1.09), p = 0.61 | 1.03 (0.98, 1.08), p = 0.44 | 0.97 (0.93, 1.02), p = 0.55 | 0.96 (0.91, 1.0), p = 0.33 | 0.97 (0.86, 1.09), p = 0.85 |
| General health | Self-rated health: -1 rating (5-point scale, decreasing health) | **1.35 (1.11, 1.64), p = 0.02** | **1.55 (1.14, 2.1), p = 0.03** | 0.86 (0.65, 1.14), p = 0.56 | **1.3 (1.11, 1.52), p = 0.01** | 1.13 (0.94, 1.36), p = 0.55 | 1.29 (0.75, 2.24), p = 0.78 |
| General health | Self-rated health: 1. Poor | 3.02 (0.9, 10.2), p = 0.31 | **41.09 (8.36, 201.89), p < 0.0001** | Suppressed | Suppressed | 2.06 (0.71, 5.93), p = 0.55 | Suppressed |
| General health | Self-rated health: 2. Fair | **2.86 (1.49, 5.46), p = 0.01** | 2.89 (0.94, 8.83), p = 0.17 | Suppressed | **2.62 (1.41, 4.85), p = 0.01** | 1.12 (0.51, 2.43), p = 0.87 | 2.62 (0.42, 16.24), p = 0.78 |
| General health | Self-rated health: 3. Good | 1.38 (0.82, 2.32), p = 0.55 | 2.0 (0.8, 4.98), p = 0.3 | 1.14 (0.53, 2.46), p = 0.86 | **1.91 (1.17, 3.12), p = 0.05** | 1.25 (0.74, 2.11), p = 0.78 | 1.32 (0.24, 7.17), p = 0.91 |
| General health | Self-rated health: 4. Very Good | 1.01 (0.63, 1.64), p = 0.95 | 2.19 (0.89, 5.34), p = 0.21 | 0.92 (0.5, 1.72), p = 0.89 | **1.84 (1.16, 2.92), p = 0.05** | 0.99 (0.62, 1.58), p = 0.98 | 1.16 (0.27, 4.97), p = 0.96 |
| General health | Self-rated health: Poor, Fair | **2.61 (1.55, 4.38), p = 0.004** | **2.69 (1.29, 5.57), p = 0.04** | Suppressed | 1.54 (0.95, 2.49), p = 0.32 | 1.26 (0.7, 2.26), p = 0.78 | 2.1 (0.51, 8.6), p = 0.78 |
| Mental health | Anxiety (GAD-7): +1 score (21-point scale) |  |  |  |  | 1.05 (1.0, 1.1), p = 0.29 | 1.09 (0.93, 1.27), p = 0.78 |
| Mental health | Anxiety (GAD-7): Above threshold |  |  |  |  | 1.27 (0.8, 2.02), p = 0.64 | 1.89 (0.48, 7.42), p = 0.78 |
| Mental health | Anxiety (HADS) score: +1 score (21-point scale) | 0.98 (0.93, 1.02), p = 0.61 | 1.03 (0.96, 1.1), p = 0.62 | 1.03 (0.97, 1.1), p = 0.6 | 0.96 (0.93, 1.0), p = 0.16 |  |  |
| Mental health | Anxiety (HADS): Above threshold, 11+ | 0.8 (0.5, 1.28), p = 0.63 | 1.28 (0.61, 2.71), p = 0.66 | 1.38 (0.75, 2.54), p = 0.56 | 0.71 (0.46, 1.11), p = 0.4 |  |  |
| Mental health | Anxiety (HADS): Above threshold, 8-10 | 0.65 (0.4, 1.06), p = 0.31 | 1.17 (0.6, 2.29), p = 0.74 | 0.62 (0.3, 1.27), p = 0.44 | 0.71 (0.47, 1.06), p = 0.34 |  |  |
| Mental health | Depression (HADS): +1 score (21-point scale) | 1.02 (0.97, 1.07), p = 0.65 | 1.04 (0.97, 1.12), p = 0.48 | 1.05 (0.98, 1.11), p = 0.44 | 0.99 (0.94, 1.03), p = 0.84 |  |  |
| Mental health | Depression (HADS): Above threshold, 11+ | 0.98 (0.55, 1.74), p = 0.95 | 0.61 (0.18, 2.1), p = 0.61 | 1.57 (0.8, 3.08), p = 0.44 | 0.88 (0.49, 1.56), p = 0.87 |  |  |
| Mental health | Depression (HADS): Above threshold, 8-10 | 0.91 (0.55, 1.5), p = 0.84 | 1.35 (0.64, 2.86), p = 0.61 | 0.55 (0.22, 1.34), p = 0.44 | 0.8 (0.52, 1.25), p = 0.73 |  |  |
| Mental health | Depression (SMFQ): +1 score (26-point scale) |  |  |  |  | 1.05 (1.0, 1.11), p = 0.33 | 1.06 (0.9, 1.26), p = 0.78 |
| Mental health | Depression (SMFQ): Above threshold |  |  |  |  | 1.74 (0.68, 4.42), p = 0.59 | Suppressed |
| Socio-demographics | Age: +1 year | 0.99 (0.97, 1.01), p = 0.61 | 1.02 (1.0, 1.04), p = 0.12 | 1.0 (0.97, 1.03), p = 0.96 | 1.01 (1.0, 1.02), p = 0.62 | 0.94 (0.89, 0.99), p = 0.14 | 1.04 (0.93, 1.16), p = 0.78 |
| Socio-demographics | Age: 18-49 |  |  |  |  |  | Suppressed |
| Socio-demographics | Age: 40-49 |  |  |  |  | Suppressed |  |
| Socio-demographics | Age: 50-59 | 0.83 (0.48, 1.46), p = 0.72 | 1.06 (0.25, 4.4), p = 0.95 | 0.79 (0.42, 1.5), p = 0.7 | 1.02 (0.55, 1.89), p = 0.95 |  |  |
| Socio-demographics | Age: 60-69 | 0.7 (0.39, 1.26), p = 0.55 | 3.81 (1.3, 11.13), p = 0.07 | 0.79 (0.35, 1.77), p = 0.77 | 0.95 (0.55, 1.65), p = 0.92 | 0.76 (0.52, 1.13), p = 0.55 | 2.72 (0.87, 8.49), p = 0.78 |
| Socio-demographics | Age: 70-79 | 0.72 (0.32, 1.61), p = 0.65 | 3.14 (1.12, 8.78), p = 0.11 | 0.93 (0.3, 2.89), p = 0.95 | 1.12 (0.68, 1.86), p = 0.87 | Suppressed | 0.41 (0.03, 5.54), p = 0.78 |
| Socio-demographics | Age: 80+ | Suppressed | 2.15 (0.68, 6.76), p = 0.36 | Suppressed | 1.07 (0.56, 2.07), p = 0.92 |  |  |
| Socio-demographics | Deprivation (IMD): Middle 40% (decile 4-7) | 0.73 (0.49, 1.08), p = 0.39 | 1.88 (1.04, 3.37), p = 0.11 | 1.15 (0.66, 2.01), p = 0.82 | 1.08 (0.78, 1.5), p = 0.87 |  |  |
| Socio-demographics | Deprivation (IMD): Most deprived 30% (decile 1-3) | 1.29 (0.73, 2.28), p = 0.63 | 2.45 (0.97, 6.21), p = 0.17 | 1.21 (0.55, 2.68), p = 0.82 | 0.83 (0.46, 1.53), p = 0.87 |  |  |
| Socio-demographics | Deprivation (IMD): Most deprived 40% (decile 1-4) | 1.49 (0.94, 2.36), p = 0.31 | 1.29 (0.61, 2.74), p = 0.66 | 0.97 (0.51, 1.84), p = 0.96 | 1.19 (0.78, 1.8), p = 0.79 | 0.87 (0.54, 1.4), p = 0.81 | 0.6 (0.13, 2.63), p = 0.78 |
| Socio-demographics | Employment status: In education | Suppressed | Suppressed | Suppressed | Suppressed | Suppressed |  |
| Socio-demographics | Employment status: Looking after home or family (unpaid care) | 0.92 (0.34, 2.48), p = 0.94 | Suppressed | 1.66 (0.43, 6.37), p = 0.7 | 0.79 (0.28, 2.2), p = 0.87 | 0.81 (0.28, 2.36), p = 0.87 | 7.87 (0.12, 520.99), p = 0.78 |
| Socio-demographics | Employment status: Maternity leave |  |  | Suppressed |  |  |  |
| Socio-demographics | Employment status: Other | Suppressed | Suppressed | Suppressed | Suppressed |  |  |
| Socio-demographics | Employment status: Permanently (or long-term) sick or disabled | 2.64 (0.69, 10.13), p = 0.47 | **13.66 (2.25, 83.13), p = 0.03** | Suppressed | 1.41 (0.41, 4.91), p = 0.87 | Suppressed | Suppressed |
| Socio-demographics | Employment status: Retired | 1.4 (0.83, 2.36), p = 0.55 | 2.73 (0.9, 8.29), p = 0.2 | 0.85 (0.3, 2.48), p = 0.89 | 1.05 (0.63, 1.74), p = 0.92 | 0.87 (0.51, 1.48), p = 0.85 | 0.51 (0.08, 3.12), p = 0.78 |
| Socio-demographics | Employment status: Self-employed | 1.53 (0.88, 2.64), p = 0.41 | Suppressed | 0.72 (0.28, 1.84), p = 0.7 | 0.86 (0.4, 1.85), p = 0.91 | 1.23 (0.71, 2.14), p = 0.78 | 0.74 (0.08, 7.03), p = 0.94 |
| Socio-demographics | Employment status: Semi-retired/part-time employment | Suppressed | Suppressed | Suppressed | Suppressed |  |  |
| Socio-demographics | Employment status: Unemployed | Suppressed |  | Suppressed | Suppressed | 1.22 (0.35, 4.33), p = 0.87 | Suppressed |
| Socio-demographics | Employment status: Unpaid/voluntary work | Suppressed | Suppressed | Suppressed | 0.94 (0.31, 2.84), p = 0.95 | Suppressed | Suppressed |
| Socio-demographics | Ethnicity: Other than white | Suppressed | Suppressed | Suppressed | 1.76 (0.66, 4.7), p = 0.62 | Suppressed | Suppressed |
| Socio-demographics | IMD: -1 decile (increasing deprivation) | 1.03 (0.95, 1.11), p = 0.65 | 1.11 (0.99, 1.24), p = 0.18 | 1.02 (0.93, 1.12), p = 0.85 | 0.99 (0.93, 1.06), p = 0.92 |  |  |
| Socio-demographics | IMD: -1 quintile (increasing deprivation) |  |  |  |  | 1.0 (0.85, 1.17), p = 0.98 | 0.87 (0.53, 1.45), p = 0.86 |
| Socio-demographics | Highest educational attainment: NVQ level 3 or lower | 0.88 (0.59, 1.32), p = 0.72 | **2.23 (1.26, 3.94), p = 0.03** | 1.09 (0.6, 1.98), p = 0.89 | 1.03 (0.74, 1.44), p = 0.92 | 1.42 (0.93, 2.18), p = 0.48 | 1.02 (0.25, 4.15), p = 0.99 |
| Socio-demographics | RUC: Urban | 1.01 (0.68, 1.51), p = 0.95 | 0.88 (0.49, 1.61), p = 0.78 | 0.5 (0.29, 0.87), p = 0.05 | 1.16 (0.8, 1.66), p = 0.79 |  |  |
| Socio-demographics | Sex: Male | 1.4 (0.86, 2.27), p = 0.49 | 0.64 (0.26, 1.58), p = 0.55 | 1.06 (0.5, 2.25), p = 0.93 | 0.79 (0.47, 1.35), p = 0.77 | 1.62 (1.1, 2.37), p = 0.13 | 1.99 (0.56, 7.04), p = 0.78 |
