# Supplementary material for: Antibody levels following vaccination against SARS-CoV-2: associations with post-vaccination infection and risk factors in two UK longitudinal studies
Source: eLife. 2023 Jan 24;12:e80428. doi: 10.7554/eLife.80428 (PMC9940912; doi:10.7554/eLife.80428)
Supplement: Supplementary file 8. — Pair differences are calculated between all complete pairs of monozygotic (MZ) twins and/or dizygotic (DZ) twins, and all combinations of non-related pairs. [file elife-80428-supp8.docx]

Supplementary file 8. Descriptive statistics of differences in anti-Spike antibody levels between pairs after third SARS-CoV-2 vaccination within TwinsUK. Pair-differences are calculated between all complete pairs of monozygotic (MZ) twins and/or dizygotic (DZ) twins, and all combinations of non-related pairs.

|  | **Intra-pair difference in anti-Spike levels (BAU/mL) after third vaccination** | | | |
| --- | --- | --- | --- | --- |
|  | **Difference between non-related pairs** | **Difference within related pairs** | **Difference within DZ pairs** | **Difference within MZ pairs** |
| **Count of pair differences** | 1874561 | 455 | 167 | 286 |
| **Mean** | 8744 | 7037 | 8061 | 6444 |
| **Standard deviation** | 6369 | 5735 | 6112 | 5441 |
| **Minimum** | 0 | 0 | 0 | 0 |
| **25%** | 3205 | 2332 | 2770.25 | 1984 |
| **Median** | 7862 | 5678 | 6831 | 4981 |
| **75%** | 13546 | 10934 | 12481 | 9715 |
| **Maximum** | 25000 | 24625 | 23189 | 24625 |
