# Supplementary material for: Antibody levels following vaccination against SARS-CoV-2: associations with post-vaccination infection and risk factors in two UK longitudinal studies
Source: eLife. 2023 Jan 24;12:e80428. doi: 10.7554/eLife.80428 (PMC9940912; doi:10.7554/eLife.80428)
Supplement: Supplementary file 9. — Coefficients with unadjusted 95% confidence intervals and unadjusted p-values are presented. Family structure is included as a random effect, allowing intercepts to vary between twin-pairs. Models are adjusted for age, sex, weeks since third vaccination, third vaccine received, and serology-based infection status. Variables with (two-sided) p-values <0.05 are highlighted in bold. [file elife-80428-supp9.docx]

Supplementary file 9. Results of generalised linear mixed effects models testing association with anti-Spike antibody levels after third SARS-CoV-2 vaccination within and between twin-pairs within TwinsUK. Coefficients with unadjusted 95% confidence intervals and unadjusted p-values are presented. Family structure is included as a random effect, allowing intercepts to vary between twin-pairs. Models are adjusted for age, sex, weeks since third vaccination, third vaccine received and serology-based infection status. Variables with (two-sided) p-values < 0.05 are highlighted in bold.

| **Exposure variable** | **Self-rated health (ordinal 1-5 scale, unit: +1, increasing health)** | | **Advised on "Shielded Patient List" (0: No, 1: Yes)** | | **Comorbidity: Rheumatoid arthritis (0: No, 1: Yes)** | | **Prescribed immunosuppressant medication (0: No, 1: Yes)** | | **Highest educational attainment (0: NVQ level 3 or lower, 1: NVQ level 4 or higher)** | | **Frailty index (log-transformed, unit: +1 standard deviation, increasing frailty)** | | **Multimorbidity (0: < 3/5 comorbidities, 1: 3/5 comorbidities)** | |
| --- | --- | --- | --- | --- | --- | --- | --- | --- | --- | --- | --- | --- | --- | --- |
| **Zygosity** | **MZ** | **DZ** | **MZ** | **DZ** | **MZ** | **DZ** | **MZ** | **DZ** | **MZ** | **DZ** | **MZ** | **DZ** | **MZ** | **DZ** |
| **n** | 554 | 322 | 572 | 334 | 572 | 334 | 572 | 334 | 572 | 334 | 486 | 308 | 572 | 334 |
| **Exposure variable: Family mean ('between-pair')** | **2628.02 (1958.61, 3297.44), p < 0.0001** | **1112.97 (14.1, 2211.83), p = 0.05** | -1835.79 (-5199.34, 1527.77), p = 0.28 | 1186.88 (-2790.16, 5163.91), p = 0.56 | -7410.35 (-16435.28, 1614.58), p = 0.11 | **-7066.57 (-13662.52, -470.61), p = 0.04** | -1520.6 (-4845.39, 1804.2), p = 0.37 | -3400.74 (-7132.33, 330.85), p = 0.07 | **3021.69 (1416.43, 4626.94), p = 0.0002** | 1126.5 (-880.9, 3133.89), p = 0.27 | **-1492.75 (-2408.54, -576.96), p = 0.001** | -488.47 (-1761.03, 784.09), p = 0.45 | 2243.69 (-9226.11, 13713.48), p = 0.7 | -5830.71 (-14130.41, 2469.0), p = 0.17 |
| **Exposure variable: Individual difference to family mean ('within-pair')** | 643.13 (-316.09, 1602.35), p = 0.19 | -475.4 (-1648.82, 698.03), p = 0.43 | **-3684.38 (-6491.81, -876.96), p = 0.01** | -2975.43 (-6816.68, 865.83), p = 0.13 | **-5799.45 (-11176.03, -422.86), p = 0.03** | -2842.12 (-9956.15, 4271.91), p = 0.43 | -1733.37 (-4088.0, 621.26), p = 0.15 | -1494.04 (-4518.44, 1530.36), p = 0.33 | -96.53 (-2067.99, 1874.94), p = 0.92 | -1747.06 (-4260.26, 766.15), p = 0.17 | 92.9 (-927.72, 1113.53), p = 0.86 | -873.85 (-2223.79, 476.1), p = 0.2 | -6026.79 (-12830.25, 776.68), p = 0.08 | 2646.79 (-4510.21, 9803.79), p = 0.47 |
| ***Wald test of ‘between-pair’ = ‘within-pair’*** | **p =0.0008** | p = 0.05 | p = 0.41 | p = 0.14 | p = 0.76 | p = 0.39 | p = 0.92 | p = 0.44 | **p = 0.02** | p = 0.08 | **p = 0.02** | p = 0.68 | p = 0.22 | p = 0.13 |
| **Age (unit: +1 year)** | **116.95 (78.99, 154.91), p < 0.0001** | **196.14 (133.23, 259.06), p < 0.0001** | **244.21 (222.03, 266.4), p < 0.0001** | **253.43 (224.45, 282.41), p < 0.0001** | **243.07 (220.96, 265.18), p < 0.0001** | **258.87 (229.88, 287.86), p < 0.0001** | **245.37 (222.57, 268.18), p < 0.0001** | **259.64 (230.14, 289.14), p < 0.0001** | **222.48 (198.07, 246.89), p < 0.0001** | **249.03 (218.2, 279.86), p < 0.0001** | **241.09 (217.23, 264.96), p < 0.0001** | **256.93 (227.48, 286.39), p < 0.0001** | **242.76 (220.61, 264.9), p < 0.0001** | **255.68 (226.61, 284.74), p < 0.0001** |
| **Sex: Male (reference: Female)** | -1206.07 (-3313.35, 901.22), p = 0.26 | 172.32 (-3254.85, 3599.5), p = 0.92 | -994.16 (-3241.51, 1253.19), p = 0.39 | 101.98 (-3402.5, 3606.45), p = 0.95 | -943.01 (-3180.95, 1294.92), p = 0.41 | -118.75 (-3582.72, 3345.21), p = 0.95 | -1027.05 (-3283.86, 1229.76), p = 0.37 | -120.4 (-3598.88, 3358.08), p = 0.95 | -937.44 (-3136.95, 1262.07), p = 0.4 | 51.9 (-3451.63, 3555.42), p = 0.98 | -1097.0 (-3670.17, 1476.16), p = 0.4 | -1225.65 (-5250.07, 2798.78), p = 0.55 | -971.71 (-3235.26, 1291.83), p = 0.4 | 2.67 (-3480.8, 3486.13), p = 1.0 |
| **Weeks since third vaccination (unit: +1 week)** | **-760.4 (-975.36, -545.44), p < 0.0001** | **-825.15 (-1112.87, -537.44), p < 0.0001** | **-577.95 (-793.54, -362.37), p < 0.0001** | **-831.28 (-1123.09, -539.47), p < 0.0001** | **-575.56 (-790.64, -360.48), p < 0.0001** | **-839.98 (-1127.12, -552.84), p < 0.0001** | **-593.74 (-809.02, -378.46), p < 0.0001** | **-814.6 (-1102.88, -526.31), p < 0.0001** | **-626.04 (-840.02, -412.07), p < 0.0001** | **-843.02 (-1131.61, -554.43), p < 0.0001** | **-626.13 (-865.16, -387.11), p < 0.0001** | **-825.41 (-1124.41, -526.41), p < 0.0001** | **-587.59 (-803.05, -372.13), p < 0.0001** | **-818.93 (-1108.37, -529.48), p < 0.0001** |
| **Third vaccine: mRNA-1273 (reference: BNT162b2)** | **3763.01 (1795.99, 5730.04), p = 0.0002** | 2770.26 (-225.02, 5765.53), p = 0.07 | **5016.49 (3067.23, 6965.74), p < 0.0001** | **3256.26 (286.13, 6226.39), p = 0.03** | **5235.92 (3283.09, 7188.75), p < 0.0001** | **3051.53 (98.1, 6004.97), p = 0.04** | **5150.06 (3190.12, 7110.0), p < 0.0001** | **3396.55 (439.76, 6353.35), p = 0.02** | **4778.81 (2825.59, 6732.04), p < 0.0001** | **2988.59 (6.4, 5970.77), p = 0.05** | **5117.66 (2870.42, 7364.89), p < 0.0001** | 2621.63 (-473.77, 5717.04), p = 0.1 | **5064.95 (3107.49, 7022.41), p < 0.0001** | **3332.52 (362.72, 6302.31), p = 0.03** |
| **Third vaccine: Other (reference: BNT162b2)** | -2958.22 (-7800.03, 1883.59), p = 0.23 | 4712.73 (-2337.57, 11763.03), p = 0.19 | -2437.17 (-7369.43, 2495.09), p = 0.33 | 4996.75 (-2140.9, 12134.4), p = 0.17 | -2700.36 (-7632.39, 2231.67), p = 0.28 | 4623.3 (-2496.36, 11742.96), p = 0.2 | -2366.81 (-7326.11, 2592.5), p = 0.35 | 5062.52 (-2076.14, 12201.17), p = 0.16 | -2640.2 (-7575.46, 2295.06), p = 0.29 | 4859.49 (-2270.14, 11989.11), p = 0.18 | -2384.03 (-7411.74, 2643.69), p = 0.35 | 4693.79 (-2381.22, 11768.79), p = 0.19 | -2649.17 (-7592.78, 2294.44), p = 0.29 | 4743.8 (-2399.78, 11887.37), p = 0.19 |
| **SARS-CoV-2 infection status (serology-based): Evidence of natural infection (reference: No evidence)** | **2871.98 (1569.76, 4174.2), p < 0.0001** | **4798.46 (2800.73, 6796.18), p < 0.0001** | **3592.85 (2285.58, 4900.11), p < 0.0001** | **4883.75 (2869.54, 6897.95), p < 0.0001** | **3583.58 (2275.31, 4891.86), p < 0.0001** | **4893.27 (2907.17, 6879.36), p < 0.0001** | **3593.19 (2276.94, 4909.45), p < 0.0001** | **4803.36 (2811.87, 6794.85), p < 0.0001** | **3683.59 (2375.91, 4991.26), p < 0.0001** | **4825.56 (2823.54, 6827.59), p < 0.0001** | **3585.43 (2133.75, 5037.12), p < 0.0001** | **4494.55 (2415.7, 6573.4), p < 0.0001** | **3495.63 (2182.19, 4809.08), p < 0.0001** | **4904.14 (2911.29, 6896.99), p < 0.0001** |
| **Group variance** | **0.68 (0.4, 0.95), p < 0.0001** | 0.14 (-0.06, 0.35), p = 0.17 | **0.89 (0.56, 1.23), p < 0.0001** | 0.16 (-0.05, 0.37), p = 0.13 | **0.88 (0.55, 1.2), p < 0.0001** | 0.14 (-0.06, 0.34), p = 0.17 | **0.88 (0.55, 1.2), p < 0.0001** | 0.15 (-0.06, 0.35), p = 0.16 | **0.81 (0.5, 1.12), p < 0.0001** | 0.16 (-0.05, 0.37), p = 0.13 | **0.68 (0.38, 0.98), p < 0.0001** | 0.21 (-0.02, 0.44), p = 0.07 | **0.89 (0.56, 1.22), p < 0.0001** | 0.15 (-0.05, 0.35), p = 0.15 |
